# Deep learning in acute ischemic stroke imaging: a systematic review of CT- and MRI-based segmentation, triage, and prognostic modeling

**DOI:** 10.1007/s00234-026-04095-5

**Published:** 2026-06-26

**Authors:** Ismail Kunduracioglu, Suat Ince, Bilal Bayram, Serhat Kilicarslan, Erkan Veziroglu, Ishak Pacal

**Affiliations:** 1 Department of Computer Engineering, Igdir University, Igdir, Turkey; 2 Department of Radiology, Van Education and Research Hospital, Van, Turkey; 3 The Department of Neurology, Van Education and Research Hospital, Van, Turkey; 4 Department of Software Engineering, Bandırma Onyedi Eylül University, Bandirma, Turkey; 5 Department of Computer Technologies, Dumlupinar University, Kütahya, Turkey; 6 Department of Electronics and Information Technologies, Nakhchivan State University, Nakhichivan, Azerbaijan; 7 Department of Computer Engineering, Faculty of Engineering and Natural Sciences, Fenerbahce University, Istanbul, Turkey

**Keywords:** Acute ischemic stroke, Brain lesion segmentation, Deep learning, Computed tomography, Magnetic resonance imaging, U-net and variants

## Abstract

**Background:**

Acute ischemic stroke is a time-critical neurological emergency in which imaging directly influences diagnosis, treatment eligibility, tissue-at-risk estimation, workflow prioritization, and outcome prediction. Deep learning has become increasingly prominent in stroke imaging; however, its clinical role differs substantially between computed tomography (CT) and magnetic resonance imaging (MRI).

**Objective:**

This PRISMA-guided systematic review evaluated peer-reviewed studies published between January 2023 and March 2026 that applied deep learning to acute ischemic stroke imaging.

**Methods:**

Searches across major biomedical, engineering, and clinical databases identified 91 eligible studies. Extracted data included imaging modality, dataset source, clinical task, model architecture, validation strategy, and reported performance metrics.

**Results:**

MRI-based studies predominantly addressed lesion segmentation and tissue characterization using DWI, ADC, FLAIR, and multimodal MRI. In this setting, U-Net-derived architectures, self-configuring frameworks, attention-based models, and selected CNN-Transformer hybrids frequently achieved strong Dice and IoU values on benchmark datasets, particularly ISLES 2015 and ISLES 2022. CT-based studies followed a different clinical direction, with greater emphasis on emergency triage, hemorrhage exclusion, large-vessel occlusion detection, stroke classification, ASPECTS-related assessment, and CTP-based core-penumbra estimation. High retrospective accuracy or AUC values in CT studies should be interpreted cautiously, since image-level classification performance does not establish reliable voxel-level lesion delineation, especially on NCCT, where early ischemic changes remain subtle and difficult to segment.

**Conclusion:**

Across both modalities, technical progress remains ahead of clinical validation. External testing, patient-level separation, standardized reporting, scanner robustness, interpretability, and prospective workflow evaluation are still inconsistently addressed. The field now requires a shift from isolated benchmark performance toward clinically interpretable, externally validated, and prognosis-aware systems that can support real-time neuroradiology decision-making.

## Introduction

Ischemic stroke remains a leading cause of mortality and long-term disability worldwide. Approximately 15 million individuals suffer a stroke annually, and of these, five million die while another five million are left with permanent damage [1, 2]. Stroke ranks second among all causes of death and lost ability, accounting for a significant portion of the 17.9 million vascular disease-related deaths recorded each year [3]. The burden is disproportionately borne by poorer nations, given that 7 out of every 10 new strokes, totaling 11.9 million cases, occur in low- or middle-income countries. Furthermore, the disease is increasingly affecting younger demographics; notably, one in three people who die from stroke has not yet reached 70 years of age [4].

Roughly 87% of strokes are ischemic strokes - a clot blocks a brain vessel [5]. Treatment of Acute Ischemic Stroke must start within minutes - the phrase “Time is Brain” fixes the clinical priority [6]. After the vessel closes, damage spreads for minutes to days [7]. Dead tissue forms the ischemic core - around it lies the penumbra - cells that still receive some blood, work poorly - yet remain rescuable [8]. Saving the penumbra is the main aim of stroke care, achieved chiefly - reopening the artery. For large vessel blockage, Endovascular Thrombectomy (EVT) is now routine and strongly raises the chance of a good result [9]. Intravenous alteplase (rtPA) is still the reference thrombolytic plus is given first in many patients [10], as shown by NINDS besides ECASS III [11, 12]. Yet angiography guided thrombectomy has limits - tiny clots can remain downstream or micro circulation can stay blocked after the main artery is open - tissue perfusion fails and prognosis worsens [6]. This shortfall demands faster, exact diagnostics and extra treatments [13].

Neuroimaging forms the core of diagnosis. A plain Computed Tomography (CT) scan without contrast remains the first test, used chiefly to rule out bleeding [14]. Magnetic Resonance Imaging (MRI), by contrast, detects early signs of ischemia far better - its Diffusion-Weighted sequence clearly shows the area that has already died [5, 7]. Yet both methods share one major hurdle - a human must trace the damaged tissue by hand. That step demands hours of expert labor - an impossible delay during an emergency - different readers often outline unlike regions [15]. Because Ischemic Strokes increase as hypertension and diabetes stay poorly controlled, the field now needs tools that scale, give consistent numbers and work fast [16].

Artificial Intelligence (AI), specifically Deep Learning (DL), has become a fundamental element of modern stroke research in this scenario. Around 2015, DL models started to acquire new ground by detecting the very intricate and detailed patterns in high-dimensional CT and MRI scans, which traditional segmentation methods fail to detect [15]. One such major milestone was the ISLES public challenge [17] which has been running from 2015 to 2024 that not only attracted researchers but also made them develop sophisticated models using attention mechanisms and multi-modal learning that can work with diffusion-weighted imaging (DWI), fluid-attenuated inversion recovery (FLAIR), and perfusion maps [18]. Nevertheless, the translation into the medical field is still plagued by several persistent limitations. A lot of models still lack the capability to be generalized across various scanners and clinics, are still too slow for the acute workflow’s real-time deployment, and are non-transparent which is a critical barrier to adoption through high-stakes clinical decision-making. The situation for example, in low-resource settings is even worse, thus aggravating the issue of global health disparities [19].

To address this gap, the present review examines deep learning studies in acute ischemic stroke imaging from a modality-specific clinical perspective. Rather than treating CT and MRI as interchangeable inputs for a single segmentation problem, we distinguish their different roles in acute care. CT-based research is frequently oriented toward rapid triage, hemorrhage exclusion, large-vessel occlusion detection, ASPECTS-related assessment, and CTP-derived core-penumbra estimation. MRI-based research, in contrast, is more often focused on precise lesion delineation, tissue characterization, and longitudinal or prognosis-oriented modeling [20]. This distinction is important because classification accuracy, AUC, Dice score, and IoU do not represent equivalent clinical endpoints. Therefore, this review synthesizes CT- and MRI-based studies separately while also evaluating how the field is moving from lesion-level segmentation toward clinically integrated prognostic modeling [21].

Although several reviews have summarized artificial intelligence applications in stroke imaging, fewer have explicitly compared CT- and MRI-based deep learning studies according to their distinct clinical tasks, imaging constraints, validation strategies, and reported metrics. The present review therefore provides a focused synthesis of recent literature by separating segmentation, classification, detection, triage, and prognosis-oriented studies. Particular attention is given to why MRI-based models often achieve stronger spatial overlap metrics for lesion segmentation, whereas CT-based models more commonly report high diagnostic classification or detection performance but continue to face challenges in NCCT lesion boundary delineation. The review also examines the emerging transition from pixel-level lesion analysis toward models that incorporate imaging and clinical information for outcome prediction, recurrence risk estimation, and treatment-oriented decision support.

## Methodology

**Fig. 1:**
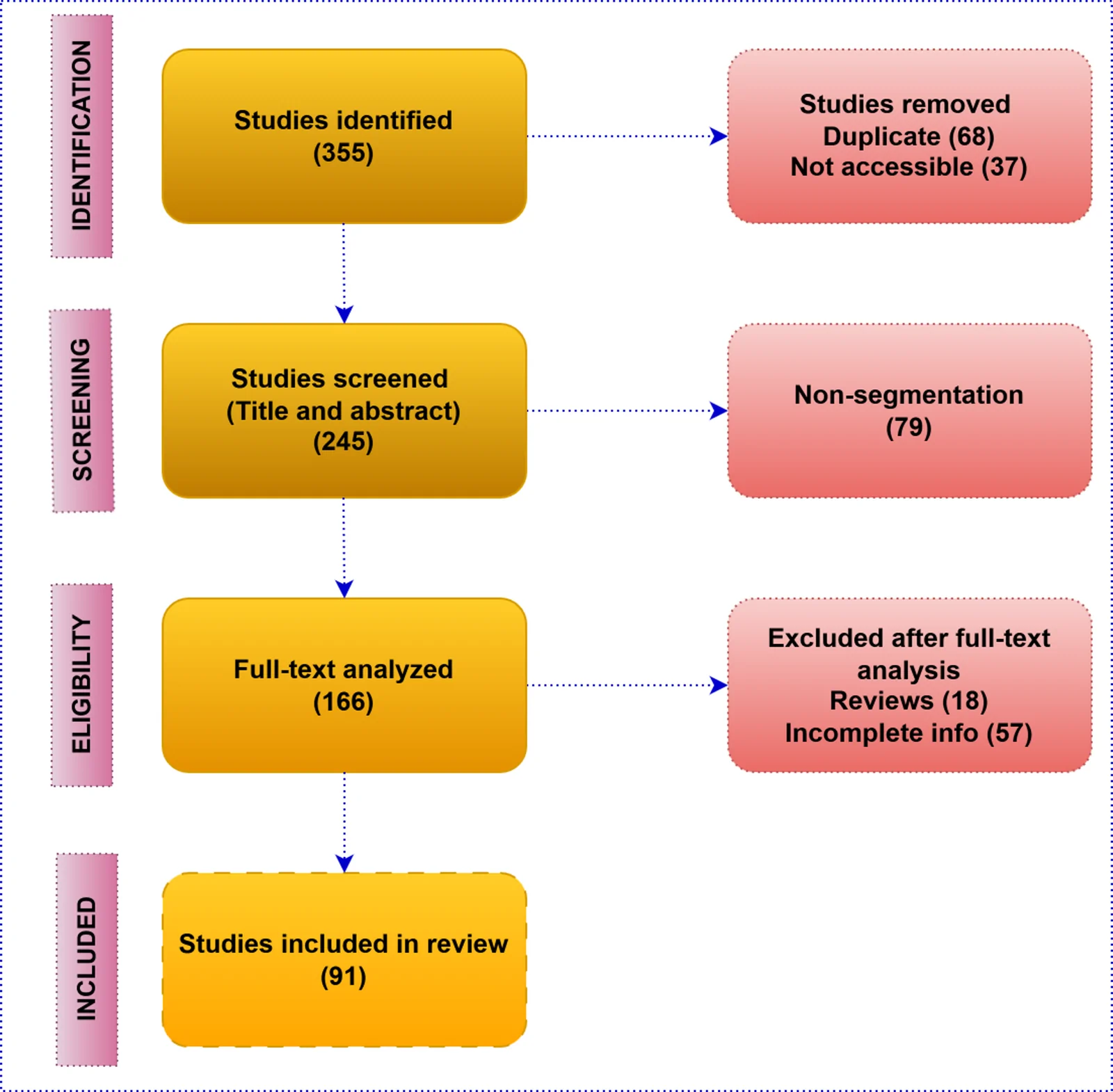
PRISMA flow diagram detailing the methodological stages of the systematic literature search and study selection process [21]

This systematic review was prepared in line with the Preferred Reporting Items for Systematic Reviews and Meta-Analyses (PRISMA) recommendations to examine recent deep learning studies in acute ischemic stroke imaging [21]. The search covered peer-reviewed articles published from January 1, 2023, to March 15, 2026. As shown in Figs. 1 and 355 records were first identified from nine electronic databases. After duplicate removal and accessibility checks, 245 records were screened by title and abstract. Of these, 166 articles were assessed in full text, and 91 original peer-reviewed studies met the eligibility criteria and were included in the final review.

### Search strategy and databases

To establish a comprehensive and systematic overview of the current state of the art, we performed an exhaustive literature search on nine prominent electronic data sources that are regularly used in the fields of medical imaging, biomedical engineering, and clinical research. The data sources searched include PubMed, Embase, Web of Science Core Collection, Scopus, IEEE Xplore, ScienceDirect, SpringerLink, Wiley Online Library, and Google Scholar. The literature search was performed for articles from 2023 to 2026 volumes or issues. This period was selected to cover the latest architectural advances, from traditional CNNs to Transformer models and their hybrids for acute stroke imaging. The comprehensive literature search was conducted between February 15, 2026, and March 15, 2026, covering articles published from January 1, 2023, up to March 2026. Following the initial database search, all retrieved citations were exported to a reference management software (e.g., EndNote), where duplicate records were automatically identified and removed. The remaining unique records were then independently screened by two reviewers (Authors A and D) based on titles and abstracts. Any disagreements during the screening phase were resolved through discussion or by consulting a third senior reviewer (Author F) to reach a consensus.

The search strategy was defined before screening and combined terms related to stroke, imaging modality, clinical task, and deep learning method. A representative search string was: (“ischemic stroke” OR “acute ischemic stroke” OR “cerebral infarction” OR “ischemic brain lesion” OR “large vessel occlusion” OR “LVO”) AND (“segmentation” OR “lesion delineation” OR “classification” OR “detection” OR “triage” OR “ASPECTS” OR “core” OR “penumbra” OR “outcome prediction” OR “prognosis”) AND (“deep learning” OR “neural network” OR “convolutional neural network” OR “CNN” OR “U-Net” OR “nnU-Net” OR “YOLO” OR “vision transformer” OR “Transformer” OR “hybrid model”) AND (“CT” OR “NCCT” OR “CTA” OR “CTP” OR “computed tomography” OR “MRI” OR “DWI” OR “ADC” OR “FLAIR” OR “perfusion imaging”). The query was adjusted for each database according to its indexing system and search syntax. This approach was used to capture both lesion-level segmentation studies and CT-based studies addressing clinically relevant triage, detection, and classification tasks.

Specific search syntaxes were adapted for each database (e.g., MeSH terms for PubMed, IEEE command line). For Google Scholar, due to the lack of advanced filtering, the first 200 results sorted by relevance were screened to identify grey literature. The final search strings were developed iteratively and subjected to internal peer review among the co-authors to ensure comprehensive coverage. To empirically validate the search strategy before the full literature retrieval, a pilot search was conducted to confirm the successful capture of a pre-defined set of seminal benchmark articles (e.g., foundational DL architectures and highly cited ISLES challenge papers). Only after this validation was the full search executed.

To ensure accessibility and reproducibility, eligibility was restricted to peer reviewed full text original research articles published in English that reported quantitative results for automatic segmentation or delineation of ischemic stroke lesions on CT or MRI using DL or closely related neural network based methods. Conference abstracts, reviews, editorials, case reports, and studies that focused exclusively on classification or detection without providing a segmentation output were excluded. In addition, the reference lists of all included studies and of recent review articles were screened manually to identify further relevant work that might not have been retrieved by the database searches.

### Inclusion and exclusion criteria

Studies were included if they investigated acute or subacute ischemic stroke imaging with CT, CTP, CTA, MRI, DWI, ADC, FLAIR, or multimodal imaging and used a deep learning or neural network-based method. Eligible studies had to report quantitative results for at least one clinically relevant task, including lesion segmentation, infarct core estimation, penumbra estimation, stroke classification, large-vessel occlusion detection, hemorrhage exclusion, ASPECTS-related assessment, or prognosis prediction. Only full-text, peer-reviewed original research articles written in English were included. Because CT is often used in the emergency setting for rapid triage rather than for detailed lesion boundary delineation alone, CT-based classification and detection studies were retained when they addressed acute stroke decision support. MRI-only studies that focused solely on simple stroke/no-stroke classification without lesion-level, tissue-level, or prognostic relevance were not included in the primary segmentation synthesis.

Conversely, the exclusion criteria were as follows: (1) Review articles, meta-analyses, editorials, or letters to the editor. (2) Conference abstracts or studies where the full text was inaccessible. (3) For MRI-based studies, research focusing solely on classification (e.g., stroke presence/absence) or detection without providing a pixel-level segmentation output was excluded. (Note: As explicitly stated in Inclusion Criterion 5, CT-based classification and detection studies were retained as an intentional exception to accurately reflect CT’s primary clinical role as a rapid triage tool, where strict segmentation is often secondary to immediate diagnosis). (4) Studies focused on the segmentation of non-lesion structures, such as cerebral arteries or carotid plaques (while these may be used for context in the introduction and discussion, they were excluded from the primary quantitative analysis).

### Data extraction and quality assessment

For each study, we extracted the publication year, imaging modality, clinical task, dataset source, sample size, public or private dataset status, model architecture, validation strategy, external validation status, reference standard, and reported performance metrics. Segmentation studies were mainly summarized using Dice similarity coefficient, IoU, precision, recall, specificity, and, when available, boundary-related metrics. Classification and detection studies were summarized using accuracy, AUC, sensitivity, specificity, precision, recall, and F1-score [22, 23].

Study quality was assessed using a structured imaging-AI framework adapted from QUADAS-2 and CLAIM recommendations. The assessment considered dataset transparency, patient-level data separation, clarity of the reference standard, external validation, appropriateness of reported metrics, and clinical applicability. Studies based only on small, single-center, private datasets without external validation were considered to have higher concern for generalizability. Studies that reported only accuracy in highly imbalanced settings were also interpreted cautiously because accuracy may overestimate model performance. These quality indicators were not used to exclude studies, but they informed the interpretation of the results and the discussion of clinical translation.

## The landscape of Ischemic stroke imaging and benchmark datasets

The overall limit to the performance of DL models in ischemic stroke lesion segmentation is ultimately determined by the quality, quantity, and diversity of the data. While, in most cases, the data is abundant for most computer vision tasks, the medical image domain is plagued by the issue of data scarcity, an issue that is heavily compounded by the very high ethical and legal hurdles that must be cleared before patient data can be utilized. The issue of obtaining informed consent is one of the most prominent hurdles to be cleared in the creation of large-scale datasets.

Furthermore, the actual annotation of data is another issue that affects the overall creation of ground-truth data. The actual creation of ground-truth data is heavily labor-intensive, as it is often performed by radiologists. Data is, therefore, the main limit to the overall performance of DL models, and the lack of diversity is one of the most prominent issues affecting the overall generalization of state-of-the-art models.

### Imaging modalities and their characteristics for segmentation

CT and MRI play different roles in acute ischemic stroke care, and this difference directly affects how deep learning models should be designed and evaluated. Non-contrast CT is usually the first imaging test in the emergency setting because it is fast, widely available, and reliable for excluding intracranial hemorrhage. CTA and CTP can add information about vessel occlusion, collateral circulation, infarct core, and potentially salvageable tissue. MRI, particularly DWI and ADC, is more sensitive to early ischemic injury and is better suited for detailed lesion delineation. For this reason, CT-based models are often evaluated as triage or detection tools, whereas MRI-based models are more commonly assessed as segmentation or tissue-characterization tools.

#### CT-based segmentation

The use of CT scanning as the gold standard imaging technique for acute ischemic stroke assessment in the clinic is well-recognized. The reasons for its popularity include the quickness of the process, its availability, and the high sensitivity for excluding bleeding in hemorrhagic stroke to provide timely treatment. Nevertheless, the segmentation of stroke-related structures on the CT scan, especially the Non-Contrast CT (NCCT) scan, is a rather complicated process. The initial manifestations of acute ischemic stroke, such as hypodensity, cytotoxic edema, and the lack of insular ribbon, are difficult to detect on NCCT because of their low-contrast nature. Segmentation of these structures is problematic in any case, including DL techniques. To solve this issue, CT Perfusion (CTP) imaging tries to give the distribution of the ischemic core and penumbra (recoverable tissue) in the form of several maps (CBF, CBV, TTP). At the same time, this type of segmentation can prove difficult because of various scanners, noises, and map differences.

#### MRI-based segmentation

MRI is currently considered the radiological gold standard technique for acute ischemic stroke assessment, and the Diffusion Weighted Imaging (DWI) is more frequently used in practice. The DWI shows cytotoxic edema (intra-cellular water restriction) with a very high contrast relative to its surroundings within minutes after the occurrence of an acute ischemic stroke. With a high signal-to-noise ratio (SNR) of DWI properties, the DWI is thus preferred for high-resolution DL-based stroke assessment [24]. Other sequences such as fluid-attenuated inversion recovery (FLAIR) are also applied in the subacute and chronic stages of the lesion characterization. The DWI-FLAIR mismatch is essential for determining the time window status. The Apparent Diffusion Coefficient (ADC), a quantification of the restricted diffusion obtained from DWI MRI sequence, excludes T2 shine-through effect. Multimodal MRI sequences, including DWI, ADC, and FLAIR images, have been incorporated into current networks as inputs for predictive purposes. However, identifying the optimum number and combination of sequences for feature extraction and classification is still an active research field.

### Public benchmark datasets

The success of DL models is directly contingent upon the availability of large, high-quality, labeled training datasets. However, in the field of medical imaging, data scarcity remains a fundamental challenge due to patient privacy concerns, high data acquisition costs, and the difficulty of expert annotation (labeling), especially in time-critical conditions such as ischemic stroke. In this context, publicly available benchmark datasets play a vital role in the development and testing of algorithms, and most importantly, for ensuring reproducibility and comparability.

In terms of ischemic stroke segmentation research, the ISLES series is considered the first academic benchmark. The first two editions of the ISLES dataset in 2015 and 2017 played an important role in establishing the necessary standards for Algorithmic comparison, with a strong emphasis on sub-acute lesion segmentation. However, the most significant editions of the ISLES dataset are ISLES 2018, which marked a paradigm shift from MRI to CT Perfusion and is thus considered the new standard for emergency room workflows, and ISLES 2022, which sought to address the issue of generalizability by creating a large dataset from multiple sources. Unlike the acute-focused ISLES dataset, the ATLAS dataset fills an important niche for rehabilitation and prognosis models by focusing on chronic lesions from patients who are more than three months post-stroke. BraTS is not an ischemic stroke dataset and should not be interpreted as a stroke-specific benchmark. Its relevance in this field is mainly related to transfer learning, since some studies use it to pretrain models on abnormal brain tissue segmentation before fine-tuning them on smaller stroke datasets. This use should be described with caution because brain tumors and ischemic infarcts differ in pathophysiology, imaging appearance, temporal evolution, and clinical meaning. In this review, BraTS was therefore considered only as an auxiliary pretraining resource. Finally, regarding CT-based studies, the Kaggle dataset is generally preferred for classification studies due to its availability and large dataset size, while the TEKNOFEST dataset provides an important resource for local researchers by offering a large dataset of around 7,000 images from a variety of sources (Table 1).

**Table 1:** Brain MRI and CT datasets: characteristics and reference citations

Dataset	Modality	Size / Content	Primary Task	Reference	Source Citation
ISLES Challenge Series (General)	MRI & CT	Variable (by year)	Acute/Sub-acute Segmentation	[17]	https://www.isles-challenge.org
ISLES 2015	MRI (Multimodal)	28 (SISS) + 30 (SPES) cases	Sub-acute Lesion & Perfusion Est.	[25]	https://www.isles-challenge.org/ISLES2015
ISLES 2018	CT Perfusion (CTP)	~ 94 scans (60 train / 34 test)	Ischemic Core & Penumbra Segmentation	[26]	https://www.isles-challenge.org/ISLES2018
ISLES 2022	MRI (DWI)	250 + multi-center patients	Acute Lesion Segmentation	[7]	https://isles22.grand-challenge.org
ISLES 2024	MRI / CT	*Varies (New Challenge)*	Acute Stroke (Generative & Seg.)	[17]	https://isles24.grand-challenge.org
ATLAS (v1.2 / v2)	MRI (T1-weighted)	304 scans (> 220 patients)	Chronic Lesion Segmentation	[27]	https://fcon_1000.projects.nitrc.org/indi/retro/atlas.html
APIS	CT (NCCT)	96 cases	Ischemic Stroke Segmentation	[28]	https://www.nature.com/articles/s41598-024-71273-x
BraTS (Brain Tumor Seg.)	MRI (Multimodal)	> 2,000 patients (2021)	“Abnormal Tissue” Learning	[29]	http://braintumorsegmentation.org/
Kaggle (Brain Stroke CT)	CT	~ 2,500 images	Classification / Detection	[30]	https://www.kaggle.com/datasets/afridirahman/brain-stroke-ct-image-dataset
TEKNOFEST	CT	6,951 images	Classification & Segmentation	[31]	https://www.kaggle.com/datasets/shuvokumarbasakbd/brain-stroke-colorized-dataset-teknofest-2021
AISD	CT (NCCT)	397 cases	Acute Ischemic Stroke Seg.	[32]	https://github.com/GriffinLiang/AISD
CPAISD	CT (NCCT)	112 cases	Ischemic Core & Penumbra Seg.	[33]	https://zenodo.org/records/10892316
SOOP	MRI (Multimodal)	~ 1,714 subjects	Outcome Prediction & Seg.	[34]	https://openneuro.org/datasets/ds004889
ICPSR	MRI (DWI)	2,888 cases	Acute Lesion Segmentation	[35]	https://www.icpsr.umich.edu/web/ICPSR/studies/38464
NeuralCUP	MRI / Clinical	Multicenter Cohort	Stroke Outcome Prediction (Prognosis)	[36]	http://neuralcup.bcblab.com

**Fig. 2:**
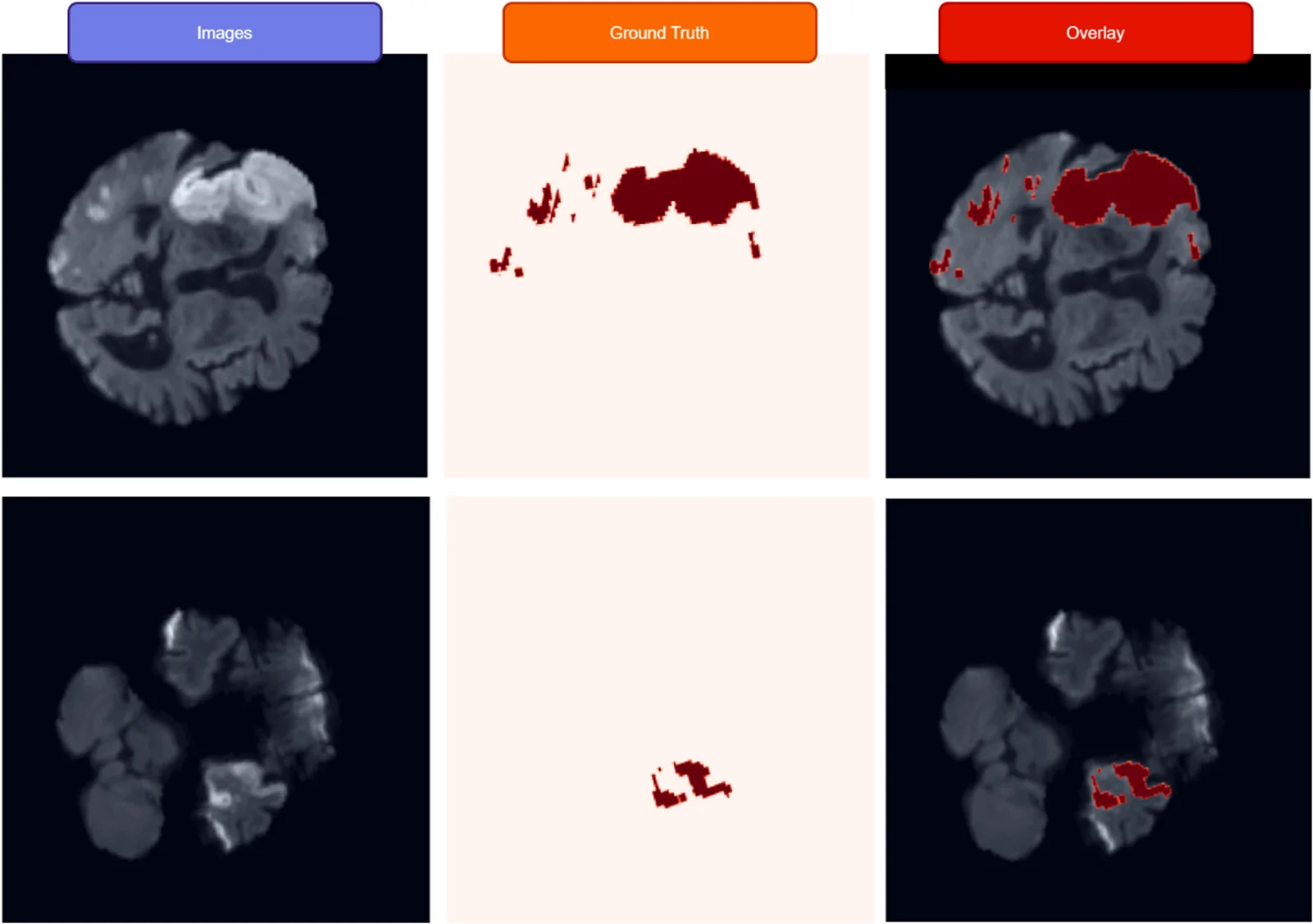
Representative MRI segmentation samples from the ISLES 2022 dataset. The columns illustrate the raw MRI input (left), expert-annotated ground truth mask (center), and the combined overlay (right), highlighting the morphological variability from large major infarctions (top row) to small focal lesions (bottom row)

**Fig. 3:**
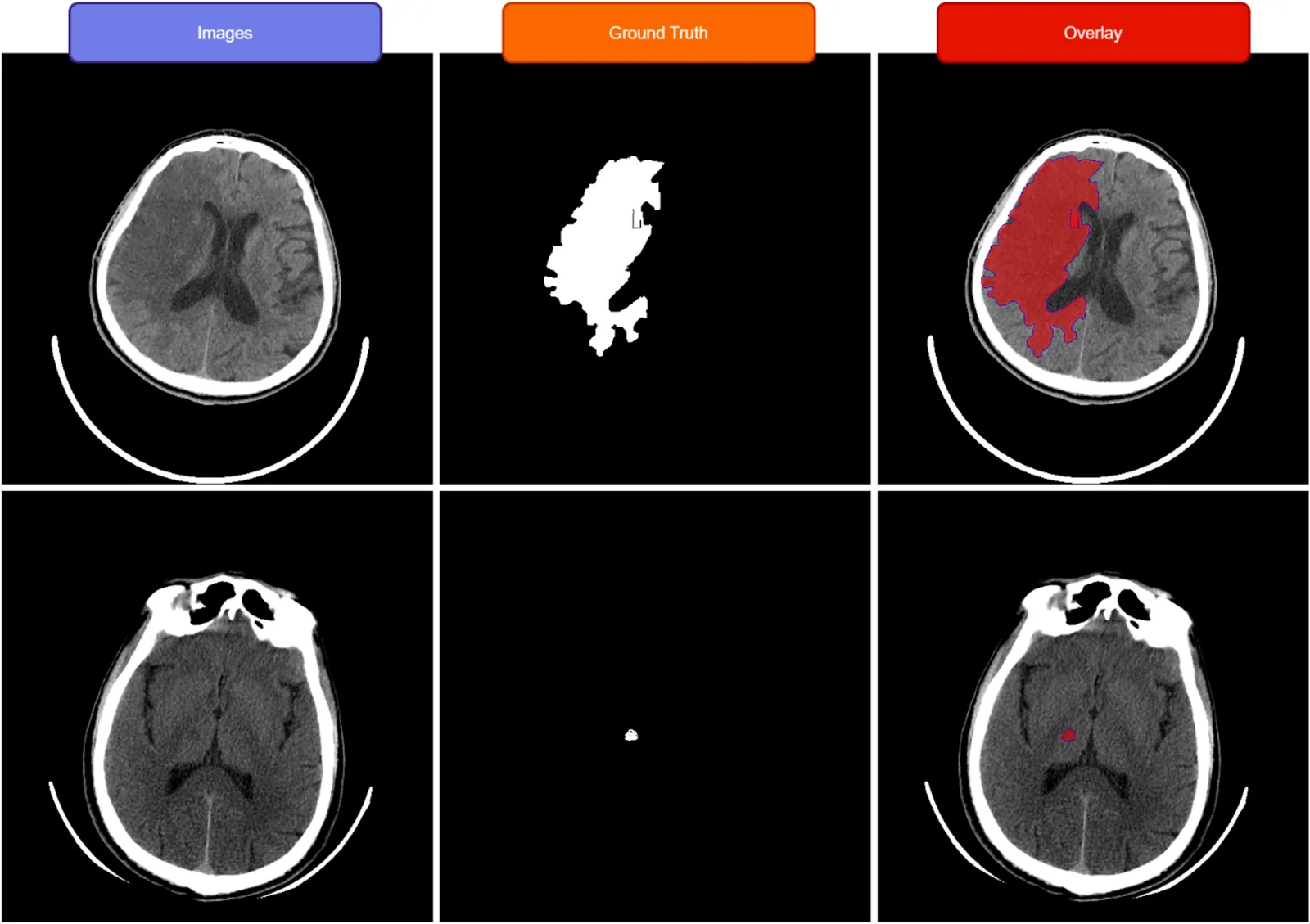
Representative CT segmentation samples from the TEKNOFEST dataset. The columns illustrate the raw CT input (left), manually delineated ground truth mask (center), and the combined overlay (right), demonstrating the clinical challenge of identifying both large (top row) and small (bottom row) ischemic lesions

To provide a qualitative insight into the segmentation tasks, representative samples from two primary benchmark datasets are visualized in Fig. 2 (ISLES 2022) and Fig. 3 (TEKNOFEST). Each figure follows a standardized three-column layout to demonstrate the relationship between the anatomical data and the pathological annotations: the first column displays the original raw input image, the second column presents the expert-annotated ground truth mask, and the third column illustrates the combined overlay where the lesion mask is superimposed onto the original scan. Furthermore, to highlight the scale invariance required from DL models, the samples are organized by lesion size; the upper rows depict cases of large volumetric lesions (e.g., Large Vessel Occlusion), whereas the lower rows demonstrate the challenging detection of small, focal lesions (e.g., lacunar infarcts). These visualizations underscore the morphological diversity and the varying degrees of difficulty inherent in both MRI and CT-based stroke segmentation.

#### ISLES (Ischemic stroke lesion segmentation)

The ISLES challenge series, unequivocally, represents the most salient and widely used academic benchmark data set in this particular domain.

##### ISLES 2015 and 2017

The inaugural ISLES challenges, held in conjunction with the MICCAI conference, focused on the MRI modality. ISLES 2015 comprised two sub-challenges: SISS (Sub-Acute Ischemic Stroke Segmentation) offering multimodal MRI data (FLAIR, T1, T2, DWI) from 28 patients for subacute lesion segmentation, and SPES (Stroke Perfusion Estimation and Segmentation) offering multimodal data including perfusion MRI (PWI) and structural MRI data from 30 patients for core/penumbra estimation in acute stroke. ISLES 2017 built upon this, increasing the data set to 43 training and 32 testing cases. The inaugural ISLES challenges marked the first standardization in the field, allowing for a fair comparison of methods, and announced the advent of U-Net variants as the new standard in this field [25, 26].

##### ISLES 2018

This challenge represented a critical paradigm shift in the field. The focus pivoted from MRI to CT Perfusion (CTP) imaging, which is often more suitable for emergency department workflows. Participants were tasked with segmenting the ischemic core and penumbra using CTP maps (e.g., CBF, CBV, TTP). This dataset, comprising approximately 94 CTP scans (60 training, 34 testing), became the de facto standard for CTP-based segmentation algorithms and is still widely used in contemporary studies [37, 38].

##### ISLES 2022

The most recent ISLES challenge shifted the focus back to MRI, specifically DWI. This iteration, however, was designed on a much larger scale than previous challenges, explicitly targeting the problem of generalizability. Consisting of data from over 250 multi-center patients, ISLES 2022 aims to test how robust models are when applied to data originating from different scanners and acquisition protocols.

#### ATLAS (Anatomical tracings of lesions after stroke)

In contrast to the acute and subacute focus of ISLES, the ATLAS dataset concentrates on chronic ischemic stroke lesions. It contains T1-weighted MRI scans from patients imaged more than three months post-stroke, offering manually delineated lesion labels by expert neurologists across 304 MRI scans (from over 220 patients). While not directly applicable for acute segmentation models, the ATLAS dataset is critically important for two specific domains: (1) studies investigating the relationship between the anatomical location of the lesion in the brain and long-term functional outcomes (e.g., aphasia, motor loss), and (2) prognosis models that evaluate brain plasticity and rehabilitation strategies.

#### BRATs (Brain tumor segmentation)

The BRATs dataset, as its name implies, is not a ischemic stroke dataset. It was established for the segmentation of brain tumors, such as glioblastoma, from multimodal MRI (T1, T1-contrast, T2, FLAIR) images. However, its inclusion in this review is warranted for two strategic reasons:

##### Data-volume and quality

The BRATs dataset (e.g., BRATs 2021, containing over 2,000 patients) is substantially larger than available ischemic stroke datasets, including ISLES.

##### Transfer learning

Ischemic stroke and tumors can present similar challenges in MRI as “abnormal brain tissue” (e.g., heterogeneous structure, indistinct boundaries). Many studies in the literature, faced with data scarcity, first pre-train their models on the massive BRATs dataset to learn “abnormal tissue” detection [39]. Subsequently, this “informed” model is fine-tuned on smaller ISLES datasets to recognize ischemic stroke lesions. This approach often yields higher performance compared to models trained from scratch using only ischemic stroke data.

Therefore, BRATs is featured in the literature not as a primary ischemic stroke benchmark dataset, but rather as an “auxiliary” or “resource” dataset.

#### Other datasets

Beyond the primary benchmark datasets mentioned above, a significant portion of studies in the field utilizes private or in-house hospital datasets. While this practice facilitates the development of high-performance models, it is the most significant factor limiting the reproducibility of these studies. To fill this gap, more accessible datasets have also emerged:

##### Kaggle datasets

The Kaggle platform hosts various ischemic stroke datasets uploaded by researchers. For example, the “Brain Stroke CT Image Dataset” contains approximately 2,500 CT images (both hemorrhagic and ischemic) [40].

##### NCCT-focused datasets

The AISD (Acute Ischemic Stroke Dataset) [32] and CPAISD (Core-Penumbra Acute Ischemic Stroke Dataset) [33] provide crucial non-contrast CT data, addressing the scarcity of public data for the most challenging modality. Similarly, APIS (Paired CT-MRI Dataset) [28] offers a unique advantage by providing registered CT and MRI scans for the same patients, enabling paired-modality learning.

##### MRI and multimodal datasets

Recent contributions include SOOP (Stroke Outcome Optimization Project) [34], which focuses on multimodal MRI data linked to clinical outcomes. The ICPSR (Ischemic Stroke Lesion Segmentation) [35] dataset and NeuralCUP [36] also expand the available repository for diverse lesion segmentation tasks. These datasets are summarized in Table 1 to guide future algorithm development.

##### National competitions (e.g., TEKNOFEST)

National events, such as the TEKNOFEST Artificial Intelligence in Health competitions held in Turkey, provide specific and challenging datasets to strengthen the local research ecosystem. One study utilized the TEKNOFEST 2021 dataset, containing 6,951 brain CT images, to perform both classification and segmentation [31].

### Data augmentation

The effectiveness of DL models for images used in the medical field is largely dependent on the availability of large amounts of annotated data. However, the availability of data for stroke segmentation is inherently limited due to privacy concerns, the high cost of annotation, and the fact that pathological cases are relatively rare compared to healthy controls. Additionally, the portion of the brain affected by a stroke is relatively very small compared to the entire brain volume, leading to a significant class imbalance issue where the model might favor the background class. To overcome these issues and prevent overfitting of the models, data augmentation has emerged as an important aspect of the model’s training process. The data augmentation techniques can be classified into three main categories:

#### Classical and geometric data augmentation

This is the traditional first-line approach to data augmentation techniques for increasing the dataset used for training models. It involves applying affine transformations that simulate the variability of patient position and scanning protocols without affecting the semantic meaning of the lesion. As described by other authors in the literature, this involves rotating images, flipping them, scaling them up and down, and shifting them. Elastic deformations are also used to simulate anatomical variability of brain structures [41, 42].

#### Advanced synthetic augmentation (GANs)

As a further alternative to the effectiveness of geometric transformations, scholarly research increasingly employs Generative Adversarial Networks (GANs) to overcome the problem of insufficient data. Unlike conventional techniques, which alter the original image, GANs seek to learn the underlying distribution of stroke lesion data to create completely original lesion image data. GAN-based techniques have shown promising results in current scholarly research. For example, conditional GAN-based techniques were effectively used to improve the performance of segmentation. Similarly, the effectiveness of GAN-based techniques to improve the performance of the model on a private dataset was demonstrated [43, 44].

#### New paradigms (self-supervised learning)

A new trend to the problem of insufficient data focuses on the problem of labeled data. Recent self-supervised learning techniques using noise-induced models offer a solution to the problem of scarcity of labeled data. Techniques like the Hybrid Unet Transformer using Noise-induced Self-Supervision utilize the self-supervised paradigm to overcome the problem of scarcity of labeled data [38, 45].

## Deep learning architectures for ischemic stroke segmentation

The field of ischemic stroke lesion segmentation has undergone a radical transformation over the past decade, particularly with the DL revolution. Unlike traditional machine learning methods (e.g., Support Vector Machines, Random Forests), DL-based approaches, especially CNNs, have demonstrated superior performance in learning complex lesion patterns from raw imaging data due to their hierarchical and automatic feature extraction capabilities. This section will systematically review the primary DL architectures used in ischemic stroke segmentation, beginning with U-Net and its variants and extending to the latest Transformer-based hybrid models.

### The cornerstone: U-net and its variants

The dominant architecture in biomedical image segmentation is undoubtedly U-Net, introduced by Ronneberger et al. [46] Its success relies on two fundamental design features: (1) a symmetric encoder-decoder structure that captures the contextual “what” information of the image, and (2) skip connections that preserve the precise “where” information (boundary details) of the lesion. These skip connections allow the model to combine low-level, high-resolution feature maps from the encoder with high-level, semantic features from the decoder, enabling the accurate delineation of ischemic stroke lesion boundaries, which are often ambiguous.

#### 2D, 2.5D, and 3D U-net

The specific implementation of U-Net architectures depends heavily on the input data modality, which can consist of either two-dimensional slices or three-dimensional volumes. For the former case, the image data is processed on a per-slice basis. This method has low computational costs and can even use pre-trained models on 2D data (e.g., ImageNet). However, this method ignores the volumetric continuity between slices and might lead to inconsistent segmentation outcomes along the z-axis. Nevertheless, this method works well for situations where two-dimensional data is prominent, such as for the detection of the Hyperdense Artery Sign (HAS) or segmentation of lesions within a single image slice of a CT scan. On the other hand, the 3D version of the U-Net architecture takes the entire volumetric data set (e.g., a 3D MRI) as input and thus captures volumetric context between slices very accurately. However, the high GPU memory (VRAM) requirements and computational costs of 3D convolutions are major drawbacks for this architecture, particularly for high-resolution images. The 2.5D version of the U-Net architecture is an attempt to combine the 2D and 3D versions by taking the central slice and a stack of some adjacent slices (3, 5, or 7 slices) as input data. This architecture reduces the contextual limitations of the 2D version of the U-Net architecture [45, 47–49].

#### Advanced U-net models

The basic U-Net architecture is continuously being developed to overcome the specific challenges of ischemic stroke segmentation (e.g., small lesions, low-contrast boundaries, class imbalance). U-Net + + architecture re-designs the skip connections of the original U-Net into a dense and nested structure. This design allows for more flexible and efficient feature fusion between the encoder and decoder, improving feature propagation. Attention U-Net & ResUnet add two critical components to the basic U-Net: Residual Connections (from ResNet) and Attention Gates. Residual connections facilitate the training of deeper networks, while attention mechanisms enable the model to actively suppress irrelevant background regions (healthy brain tissue) and “focus” on regions critical for segmentation (the lesion). This significantly enhances performance, especially in low-contrast CTP or MRI images [50, 51]. nnU-Net (no-new-U-Net) is not a specific architectural innovation but rather an extremely powerful “framework”. When applied to any new dataset (e.g., ISLES 2022), nnU-Net is a meta-model that automatically configures (self-configuring) the data preprocessing, network topology (e.g., 2D vs. 3D U-Net selection), training parameters, and post-processing steps. It often outperforms many manually designed custom architectures, establishing it as a strong baseline and a gold standard in the field [52, 53].

### YOLO-based models

The implementation of YOLO when it comes to ischemic stroke imaging involves two consecutive steps, which include object detection and segmentation. The original YOLO model was designed as an object detection algorithm, where its main function was to detect objects, providing estimates on their position in the form of bounding boxes. In emergency cases, where time is essential for a diagnosis, such algorithms are vital since precision is not a priority anymore compared to quick identification. In the case of ischemic stroke, certain versions, such as Stroke YOLO, have been developed based on this particular structure, allowing lesions to be detected in a few seconds [40].

Although it was always considered incompatible with segmentation due to this problem of the bounding box, the network underwent a huge change in recent years, focusing on the need to solve problems at a pixel level. Indeed, the last models, namely YOLOv8-Seg and YOLOv9-Seg, solved this issue by introducing a head that allowed the generation of semantic masks besides the classic detection boxes. As such, this made YOLO a hybrid tool that not only diagnoses the image but also helps segmenting it. This is particularly important for use in clinical GUIs since, in this case, the tool would allow both lesion location and volume estimation [40, 54].

However, it is crucial to contextualize these advancements within the current validation hierarchy of the field. While these emerging models show considerable promise for rapid triage, their clinical validation remains limited compared to the established U-Net paradigm. U-Net and its derivatives have undergone rigorous, multi-center benchmarking on medical cohorts and remain the foundational gold standard for medical image segmentation. Conversely, YOLO-based architectures, originally optimized for high-contrast natural image datasets, have not yet been sufficiently validated on the highly subtle, low-contrast tissue boundaries typical of acute ischemic stroke. Consequently, future research must subject these rapid-inference models to rigorous, large-scale clinical testing before they can fully transition from promising GUI prototypes to trusted, standalone neuroradiology tools.

### The new frontier: transformer-based architectures

Transformer architectures, which revolutionized the field of Natural Language Processing (NLP), succeed in an area where CNNs inherently struggle: modeling long-range contextual dependencies. This is achieved through a “self-attention” mechanism. While CNNs are excellent at capturing local patterns (e.g., the edge of a lesion), Transformers can understand the global relationships between different parts of a single lesion or between the lesion and the dura mater.

As pure Vision Transformers (ViT) require exceptionally large datasets, the most successful applications in medical imaging have emerged as CNN-Transformer Hybrids: TransUNet model integrates a Transformer into the encoder pathway of a U-Net. HUT-NSS (Hybrid Unet Transformer) is a hybrid approach combining the advantages of CNNs and Transformers. When integrated with self-supervised learning (SSL) techniques, such as “Noise-induced Self-Supervised” (NSS), it has demonstrated high performance even with limited annotated data, offering a potential solution to the problem of medical data scarcity [38]. AFS-HCTNet (Hybrid SCNN-Transformer) adds an “Adaptive Feature Selection” module to the hybrid structure. It allows the model to dynamically decide whether to assign more weight to the local details from the CNN or the global context from the Transformer, which is particularly beneficial for low-contrast and ill-defined CT lesions [55]. Sparse Transformer utilizing “sparse” attention mechanisms to reduce the computational load of standard Transformers have also shown promising results on MRI images [56].

### Other emerging architectures

The research field is highly dynamic, and innovative architectures beyond U-Net and Transformers are being investigated to solve the specific challenges of ischemic stroke segmentation (e.g., spatial hierarchy, energy efficiency):

#### Capsule graph neural networks

Standard CNNs tend to lose the internal hierarchy and spatial relationships of objects (lesions). This approach, combining Capsule Networks (CapsNet) and Graph Neural Networks (GNN), aims to better model these complex topological and hierarchical relationships of lesions, particularly in DWI images [57].

#### Deep spiking neural networks

These event-based networks, which more closely mimic the operating principles of the human brain (neuron “spiking”), potentially offer significantly lower power consumption than standard CNNs. Models like DSPM-Net are being investigated for ischemic stroke detection on MRI images using this energy-efficient approach [58].

#### Language-guided models

One of the newest approaches involves using large language and vision models (e.g., CLIP) to guide the segmentation process with text “prompts”. Models like DCTP-Net aim to use a text input, such as “acute ischemic stroke lesion,” to help the model understand what it is looking for and thereby perform more accurate segmentation [59].

#### Novel optimized networks

Researchers are also aiming to enhance detection and segmentation performance on CT images using specially designed architectures, such as the Deep Kronecker Network (DKN) combined with novel meta-heuristic optimization algorithms like the Elk Herd Taylor Optimizer (EHTO), or using Multi-Attention Dense Networks (MAD-Net) [60, 61].

## Performance metrics and evaluation

Evaluating and comparing DL methods for ischemic stroke segmentation depends on a common set of evaluation criteria. The methodologies discussed in this paper incorporate metrics which can be classified as (1) spatial overlap metrics for assessing segmentation, (2) statistical metrics for voxel-based classification, and (3) classification threshold metrics for diagnostic performance.

### Voxel overlap metrics (DSC and IoU)

These metrics are the de facto standard for evaluating the spatial accuracy of segmentation tasks.

#### Dice similarity coefficient (DSC)

The most widely reported metric in segmentation literature. DSC measures the spatial overlap between the predicted segmentation mask (Prediction) and the expert-annotated ground truth (Ground Truth). It is defined as twice the area of the intersection divided by the sum of the areas of both masks. A DSC of 1.0 indicates a perfect, pixel-for-pixel match, while 0 indicates no overlap. It is particularly valued as it provides a balanced measure of both False Positives (over-segmentation) and False Negatives (under-segmentation).

#### Intersection over union (IoU / Jaccard Index)

IoU, which is also called the Jaccard Index, is closely related to DSC and measures the overlap between the regions. It is defined as the ratio of the intersection and union areas. It is considered to be a more punitive measure compared to DSC. A model with high scores on the IoU measure (above 0.80) indicates high spatial precision.

### Statistical validation metrics (recall, specificity, and precision)

These metrics, derived from the confusion matrix at the voxel level, provide a more granular understanding of a model’s error profile.

#### Recall (Sensitivity, Re)

These two terms are often used interchangeably in segmentation literature as they are mathematically identical (True Positive Rate). This metric measures the model’s ability to correctly identify all actual positive (lesion) pixels (TP / (TP + FN)). High sensitivity is clinically critical, as it indicates that the model successfully avoids False Negatives (i.e., it does not “miss” parts of the ischemic stroke lesion).

#### Specificity (Sp)

This metric measures the model’s ability to correctly identify all actual negative (healthy tissue) pixels (TN / (TN + FP)). High specificity indicates that the model successfully avoids False Positives (i.e., it does not incorrectly label healthy tissue as a lesion). In segmentation, this metric is less informative than Precision (due to the vast number of TNs in healthy tissue) but is crucial in overall classification tasks to avoid misdiagnosing a healthy patient.

#### Precision (P)

This metric measures the “exactness” of the model. Of all the pixels the model predicted as a lesion, precision quantifies the percentage that was correct (TP / (TP + FP)). High precision indicates that the model avoids False Positives (i.e., it does not “over-segment” or incorrectly label healthy tissue as a lesion). A high-precision model is crucial for preventing the overestimation of lesion volume, which can impact treatment decisions.

### Classification-based metrics (ACC and AUC)

These metrics are primarily used for classification tasks (e.g., “stroke vs. non-stroke,” “LVO present vs. absent”) but are sometimes reported (often misleadingly) for segmentation.

#### Accuracy (ACC)

This term is defined by the total proportion of correctly classified data points. It is computed as (TP + TN) / Total Pixels. The interpretation of accuracy is not recommended for the segmentation of ischemic stroke images owing to the high level of class imbalance. In brain image analysis, the proportion of True Negatives (TN) is found to be over 99% in any given image. This means that if the model is focused on classifying all the data points as True Negatives, the accuracy level can be found to be over 99%. However, the Dice Similarity Coefficient (DSC) is found to be 0. Hence, accuracy is not recommended for segmentation problems, although it is one of the primary accuracy measures for classification problems.

#### Area under the curve (AUC)

This term is defined by the area under the Receiver Operating Characteristic (ROC) Curve. AUC is found to be an effective measure for distinguishing between the positive and negative classes. A model with an AUC of 1.0 is considered perfect, while an AUC of 0.5 is considered an indication of random guessing. AUC is recommended over accuracy for classification problems, as emphasized in the literature reviewed.

## Systematic review results

### Evaluation of magnetic resonance imaging (MRI) studies

Studies focusing on MRI and its sub-modalities clearly demonstrate the evolution of DL architectures for both lesion segmentation and diagnostic tasks. To provide a robust analytical framework, the MRI-based literature is stratified into two primary clinical categories: (1) precise volumetric lesion segmentation and (2) diagnostic classification including holistic prognostic prediction. Studies focusing on pixel-level delineation primarily utilize U-Net variants and their advanced derivatives (Table 2), whereas research centered on diagnostic classification and the “Pixel-to-Prognosis” paradigm increasingly leverages hybrid architectures, ensembles, and outcome-oriented models (Table 3).

**Table 2:** Deep learning models for MRI-based Ischemic stroke lesion segmentation

Reference	Year	Modality	Dataset	Dataset-Size	Model	Performance Metrics				
						DSC	IoU	*P*	Sp	Re
[62]	2023	MRI	NR	NR	3D-CNN	84.3	NR	84.2	NR	84.4
[63]	2023	MRI	ISLES 2017	75 cases	U-Net	36.0*	NR	42.0*	NR	48.0
[64]	2023	MRI	Private	44 cases	U-Net + ResNet	79.23	NR	NR	NR	NR
[65]	2023	MRI	ISLES 2022	400 cases	U-Net + MLP	85.6	NR	88.3	85.4	NR
[66]	2023	MRI	ISLES 2022	400 cases	CNN based	85.6	NR	88.34	NR	85.39
[49]	2023	MRI	Private	NR	CNN based	86.51	NR	89.39	NR	81.76
[67]	2023	MRI	Private	NR	MoE-based	88.0	NR	NR	NR	NR
[68]	2023	MRI	Private, ISLES 2022	NR	GAN	83.62	NR	NR	NR	NR
[28]	2024	MRI	Private	51 cases	U-Net-based	63.0*	NR	NR	NR	NR
[69]	2023	DWI	UCLA, iCAS, DEFUSE	445 cases	U-Net	50.0	NR	NR	NR	60.0
[70]	2023	T1WI	ATLAS	229 cases	Transformer, UNet	73.7	NR	NR	NR	NR
[71]	2024	MRI	ISLES 2022	400 cases	U-Net-Based	80.14*	75.42*	NR	78.31*	NR
[27]	2024	MRI	Private	56 images	Adaptive Threshold	96.0	NR	NR	NR	NR
[60]	2024	MRI	ATLASv2	655 cases	AGFN	69.21	55.27	NR	70.81	99.77
[72]	2024	MRI	ATLAS	229 cases	FRPNet	60.16	NR	NR	NR	67.74
		MRI	ISLES 2022	400 cases	FRPNet	85.72	NR	NR	NR	84.93
[73]	2024	MRI	Private	327 cases	U-Net based	66.0*	NR	NR	81.0*	93.0*
[74]	2024	MRI	ATLAS	229 cases	DenseNet based	83.1*	NR	NR	NR	NR
[43]	2024	MRI	BraTS	220 cases	3D ResU-Net	87.3*	NR	69.60	NR	NR
[75]	2024	MRI	BraTS	220 cases	U-net based	77.18	NR	NR	NR	NR
		MRI	ISLES 2022	400 cases	U-net based	78.69	NR	NR	NR	NR
[76]	2024	MRI	ISLES 2015	114 cases	CNN based	90.00*	NR	NR	NR	87.00
[77]	2024	MRI	Private	218 cases	U-net based	71.00	NR	87.00	NR	99.00
[78]	2024	MRI	ATLASv2 + ISLES 2015	655 + 114 cases	CNN based	82.00*	69.0*	80.00*	NR	83.00*
[79]	2024	MRI	Private	93 cases	Cascade ResU-Net	83.00	NR	NR	NR	NR
[80]	2024	MRI	Private	147 cases	CGAN	85.98	NR	NR	NR	NR
[81]	2024	MRI	ISLES 2022	250 cases	MDANet	70.44*	NR	75.30*	NR	72.22*
			ISLES 2018	NR	MDANet	58.34*	NR	60.22*	NR	64.03*
[82]	2024	3D MRI	ATLASv2	655 cases	U-net based	73.00	NR	NR	NR	NR
[17]	2024	MRI	ISLES 2022	250 cases	Ensemble	82.0*	NR	NR	NR	NR
[83]	2024	FLAIR	ISLES 2015	NR	EnigmaNet	89.65	NR	NR	87.76	98.66
		DWI	ISLES 2015	NR	EnigmaNet	84.23	NR	NR	84.52	97.54
[84]	2025	Multi	ISLES 2015	64 cases	Res2U++	80.0*	NR	NR	NR	NR
[85]	2025	MRI	ISLES 2022	400 cases	Attention U-Net based	94.86	80.14	NR	96.1	95.74
[86]	2025	MRI	Private	612 cases	MRA-UNet	81.6	80.1	NR	62.0	97.0
[87]	2025	MRI	ISLES 2022	250 cases	U-Net-Based	89.99	82.23	90.72	NR	89.75
[18]	2025	MRI	ISLES 2022	250 cases	U-Net-Based	88.94	80.15	NR	NR	NR
[88]	2025	MRI	ISLES 2022	250 cases	**FA-UNet**	86.76	75.84	87.23	86.42	NR
[89]	2025	MRI	ISLES 2022	250 cases	DenseNet-Based	87.49	84.63	94.87		88.57
[90]	2025	MRI	ISLES 2015	64 cases	M^2^FNet	89.05	NR	NR	NR	NR
			ISLES 2022	250 cases	M^2^FNet	88.04	NR	NR	NR	NR
[91]	2025	DWI	ISLES 2022	250 cases	ASMM-Net	89.42	80.95	90.56	87.29	NR
[92]	2025	MRI	Private	514 cases	SegResNet	87.0*	79.0*	NR	NR	NR
[56]	2025	MRI	ISLES2022	246 cases	InceptionNeXt-based	86.33*	NR	NR	NR	NR
[93]	2026	MRI	ISLES2015	250 cases	ResUNet-DSM	72.6*	NR	NR	NR	89.2
[39]	2026	MRI	ATLAS	655 cases	MPFF-Net	55.41*	45.04*	67.14*	NR	54.61*
		MRI	BraTS2018	1151 cases	MPFF-Net	62.28*	48.65*	74.51*	NR	59.52*
[94]	2026	MRI	AIS	118 cases	MSCS-Net	88.72	79.78	86.64	NR	90.97
		MRI	ATLAS	655 cases	MSCS-Net	79.45	67.25	83.89	NR	75.60
		MRI	ISLES2022	250 cases	MSCS-Net	82.63	69.61	87.50	NR	77.31
		MRI	BraTS2018	1151 cases	MSCS-Net	90.59	82.81	91.18	NR	89.98
[55]	2026	MRI	ISLES2022	250 cases	AFS-HCTNet	79.43	NR	87.67	NR	77.38
		MRI	ATLASv2	655 cases	AFS-HCTNet	59.77	NR	70.69	NR	69.98
[95]	2026	MRI	ISLES2022	250 cases	U-net based	89.36	80.84	90.76	NR	87.04

(*) references to mean value, *NR* not reported

Studies in 2023 showed that diffusion-weighted scans are widely considered the gold standard for identifying the infarct core due to their high contrast sensitivity. However, automated segmentation can still be challenging due to potential artifacts, low resolution, or variability in lesion appearance. Because of this, Nazari-Farsani et al. [69] reached only about 50% DSC with a U-Net. And reported a similar score with an AG-DCNN. Transformer-based models started to appear during the same year. A transformer model tested and reached roughly 72.6% DSC [66]. Their hybrid transformer–CNN version performed slightly better, at about 73.7%. Soh et al. [70] also tried a transformer–U-Net design and obtained a similar result on T1-weighted images. Some of the highest scores of the year came from hybrid architectures. Wu et al. [65] combined a U-Net with an Multi-Layer Perceptron (MLP) and achieved about 85.6% DSC on the ISLES 2022 dataset. Ou et al. [67] used a Mixture-of-Experts model and reported nearly 88% DSC on a private dataset. Other groups explored CNN-based and GAN-based methods. When Gómez et al. [63] applied a standard U-Net to the ISLES 2017 dataset, the model achieved a mean DSC of 36.0%* (± 21.0), along with a precision of 42.0% (± 25.0) and a recall of 48.0% (± 29.0), which accurately reflects the inherent difficulty and high variance of that dataset. Another team combined U-Net components with ResNet blocks and obtained a DSC slightly above 79.23% on a private dataset [64]. Their results ranged widely, from around 61% to 86% DSC, depending on the dataset and approach.

The year 2024 was characterized by the establishment of the ISLES 2022 dataset as a standard benchmark and the widespread adoption of transfer learning techniques. Gui et al. [71] reported an 80.19% DSC using a U-Net-based model on ISLES 2022. In another research tested their FRPNet model on both the ATLAS dataset (60.16% DSC) and ISLES 2022 (85.72% DSC), demonstrating the difference in difficulty between the two benchmarks [72]. Sun et al. [73] reached 66.0 (± 17.0) DSC with 3D U-Net. The ATLAS dataset was also employed 69.21% DSC [60], 83.1% (± 6.4) DSC [74], and 73.00% DSC [82].

Studies utilizing the BraTS (brain tumor) dataset for transfer learning were notable: M. Sun et al. [43] achieved 87.3% (± 4.6) DSC with a 3D ResU-Net, and Jeong et al. [75] reported 77.18% DSC (BraTS) / 78.69% DSC (ISLES 2022) with a U-Net. Bal et al. [76] achieved 90.0% (± 8.0) DSC on ISLES 2015, while Ahmed et al. [78] combined the ATLAS and ISLES 2015 datasets, reporting 82.0% (± 39.0) DSC. In the pursuit of State-of-the-Art (SOTA) performance, de la Rosa et al. [17] reported an exceptional 82.0% (± 12.0) DSC on ISLES 2022 using a robust ensemble model. Sinha et al. [83] demonstrated higher performance on the FLAIR sequence (89.65% DSC) compared to DWI (84.23% DSC) using their EnigmaNet model. Other approaches of the year included those by 85.98% DSC with cGAN [80], 71.31% DSC with MDANet [81], 83.0% DSC with Cascade ResU-Net [79], and 82.60% DSC with U-Net [73]. The years 2025 and 2026 demonstrate intensified competition on the ISLES 2022 benchmark, with the 90% DSC band being the target. With a U-Net-based model [87] achieved 89.99% DSC, while with DWI-specific ASMM-Net model [91] obtained 89.42% DSC. Other notable studies in this SOTA race include ConvNeXtV2-GRN U-Net [18], 88.94% DSC, DenseNet-Based [89], 87.49% DSC, and FA-UNet [88], 86.76% DSC.

**Table 3:** Deep learning models for MRI-based stroke classification and prognostic prediction

Reference	Year	Modality	Dataset	Dataset-Size	Model	Performance Metrics				
						ACC	AUC	*P*	Sp	Re
[96]	2023	MRI	NR	NR	ResNet50 based	95.67	97.30	95.68	NR	95.67
[62]	2023	MRI	NR	NR	3D-CNN	81.9	NR	84.2	NR	84.4
[97]	2023	MRI	Private	225 cases	3D U-Net	85.2	NR	NR	NR	NR
[98]	2024	MRI	Private	NR	GCDCM	97.80	NR	94.73	98.3	90.45
[77]	2024	MRI	Private	218 cases	U-net based	87.00	NR	87.00	NR	99.00
[17]	2024	MRI	ISLES 2022	250 cases	Ensemble	97.6	NR	NR	NR	NR
[83]	2024	FLAIR	ISLES 2015	NR	EnigmaNet	97.18	NR	NR	87.76	98.66
		DWI	ISLES 2015	NR	EnigmaNet	96.17	NR	NR	84.52	97.54
[86]	2025	MRI	Private	612 cases	MRA-UNet	97.3	NR	NR	62.0	97.0
[58]	2026	MRI	The acute Ischemic Stroke MRI	1008 images	DSpM-Net	92.85	NR	91.87	NR	94.76
		MRI	Brain Stroke MRI Images dataset	750 cases	DSpM-Net	90.76	NR	89.65	NR	92.87
[99]	2026	MRI	NITRC	NR	DSPM-Net	99.3	99.0	98.5	99.2	98.3
[94]	2026	MRI	AIS	118 cases	MSCS-Net	99.85	NR	86.64	NR	90.97
		MRI	ATLAS	655 cases	MSCS-Net	99.47	NR	83.89	NR	75.60
		MRI	ISLES2022	250 cases	MSCS-Net	99.60	NR	87.50	NR	77.31
		MRI	BraTS2018	1151 cases	MSCS-Net	99.20	NR	91.18	NR	89.98

*NR *not reported

Research during 2023 examined MRI-based ischemic stroke analysis from two distinct angles. One line of work focused on identifying ischemic stroke cases. In this group [96] tested a ResNet50 model and reached an accuracy close to 96%. This result reflects how well deep feature extractors can handle this task. The second line of studies dealt with segmentation. Many teams preferred 3D-CNN frameworks because these models process full-volume data more naturally. Kim et al. [62] reported performance around 84% DSC and roughly 82% accuracy, showing a reasonable mix of boundary quality and detection stability. U-Net variants also remained influential. Matsulevits et al. [97] used a 3D U-Net for long-term outcome prediction, aiming to capture patterns linked to recovery.

During this period, research has also shifted beyond pure segmentation to focus on clinical and prognostic applications. Liu J. et al. [86] utilized MRA-UNet (81.6% DSC) for segmentation as well as for predicting 1-year recurrence risk. In a more clinically integrated approach, recent work [92] utilized a SegResNet-based DL model to segment both core infarcts and white matter hyperintensity (WMH) burdens on multi-sequence MRI. By combining these model-extracted radiologic features with clinical variables, demonstrating the practical utility of the ‘Pixel-to-Prognosis’ paradigm. Dou et al. [100] and Ding et al. [101] investigated the use of high-resolution MRI for endovascular therapy planning and prognosis. In classification tasks, achieved 97.80% ACC with their GCDCM model [98], while reported 96.0% ACC on the SWI modality using Adaptive Thresholding [27].

Architectural innovations continued in this period: hybrid and Transformer-based models such as those by Sparse Transformer [56], 86.33% (± 0.30) DSC, MSCS-Net [94], 82.63% DSC on ISLES2022, AFS-HCTNet [55], 79.43% DSC, and MPFF-Net [39] were introduced. Classification tasks maintained high accuracy rates with DSpM-Net [58], 92.85% ACC and 99.3% ACC on fMRI data [99]. Methodological and etiological studies, such as those by 72.6% (± 14.4) DSC, also contributed to the field [93]. Further architectural innovations include the ResUnet + + framework integrated with Atrous Spatial Pyramidal Pooling (ASPP) [84], which achieved a Dice coefficient of 85.0 on the ISLES 2022 dataset by efficiently capturing multi-scale contextual information. Similarly, the M2FNet architecture [90] has been proposed to aggregate salient features from multiple MRI modalities across various levels, outperforming several baseline methods in segmentation accuracy.

### Evaluation of computed tomography (CT) studies

CT-based studies reflect the critical role of computed tomography in the rapid emergency triage phase of acute ischemic stroke management. To accurately capture the distinct clinical objectives within this modality, the reviewed literature is stratified into two main categories. Research focusing on the highly challenging task of spatial lesion delineation, particularly on NCCT and CTP, is summarized in Table 4.

**Table 4:** Deep learning models for CT-based Ischemic stroke lesion segmentation

Reference	Year	Modality	Dataset	Dataset-Size	Model	Performance Metrics				
						DSC	IoU	*P*	Sp	Re
[102]	2024	NCCT	AISD	397 cases	OS-AISeg	61.96*	47.38*	70.32*	NR	60.78*
			ISLES 2018	103 cases	OS-AISeg	48.41*	33.41*	51.56*	NR	56.95*
			BraTS2019	335 patients	OS-AISeg	81.48	67.99	NR	NR	NR
[103]	2024	CT	Private	100 cases	YOLOv4	92.99	NR	NR	91.14	99.41
		CT	Private	100 cases	SSD	76.78	NR	NR	87.71	86.34
		CT	Private	100 cases	YOLACT	91.03	NR	NR	90.50	99.53
[104]	2024	NCCT	Private	197 cases	I2PC-Net	65.67*	NR	NR	NR	NR
[105]	2024	CT	Private	90 cases	U-Net based	79.00	NR	83.0	100	NR
[73]	2024	NCCT	Private	51 cases	Adaptive Thresholding based	66.0*	NR	NR	93.0	81.0
[106]	2024	NCCT	Private	172 cases	Hybrid CNN-Transformer	46.74	43.3	75.1	54.0	54.0
[47]	2024	NCCT	Private	81 hasta	3D Swin UNETR	47.0	NR	45.0	43.0	43.0
[107]	2025	CT	Private	200 images	FTL	66.65	NR	NR	NR	NR
[108]	2025	CT	Kaggle Multi	4975 images	VGG19	45.19*	NR	NR	NR	NR
[109]	2025	NCCT	Private	445 images	Ensemble Model	NR	NR	NR	88.3	87.8
[110]	2025	CT	Stroke CT dataset	600 cases	Hybrid CNN + Radiomics model	76.0	NR	NR	75.0	83.3
[111]	2025	CT	Merged dataset	1038 images	Ensemble Model	62.2	NR	NR	79.4	81.7
[59]	2025	CT	AIS CT Dataset	1,500 images	DCTP-Net	80.3*	76.2*	95.1*	NR	80.6*
[37]	2025	CT	ISLES2018	NR	Attention ResUnet	59.0	NR	62.0	NR	57.0
[54]	2025	CT	Private	6951 images	YoloV9-Seg	NR	NR	99.59	NR	98.93
[38]	2025	CT	ISLES 2018	NR	HUT-NSS	62.4	49.5	75.30	NR	58.7
[112]	2025	NCCT	ISLES 2018	103 cases	Attention U-Net	76.0	NR	81.0	72.0	72.0
[113]	2026	CT	Private	706 cases	DAP-Net	56.6	40.7	NR	NR	NR
[114]	2026	CT	AIS CT Dataset	1500 images	DINOv3-ViT-based	51.6	40.0	NR	NR	NR

(*) references to mean value, *NR* not reported

Research papers that were published after 2023 indicated that ischemic stroke-imaging studies had quite a consistent growth in terms of several analytical areas tackled. Wang et al. [102] through their study, on the AISD dataset (397 cases), the model achieved a mean DSC of 61.96% (± 17.86) and an IoU of 47.38% (± 19.37), alongside a precision of 70.32% (± 19.19) and a recall of 60.78% (± 21.53). When applied to the ISLES 2018 dataset (103 cases), which presents its own complexities, it yielded a mean DSC of 48.41% (± 18.01), an IoU of 33.41% (± 15.81), a precision of 51.56% (± 24.43), and a recall of 56.95% (± 25.31). Furthermore, on the BraTS 2019 dataset (335 patients), the model demonstrated strong performance with a mean DSC of 81.48% and an IoU of 67.99%.

Segmentation studies revealed a wide range of results: Michaliszen et al. [103] achieved 92.99% DSC with YOLOv4, Kuang et al. [104] reported 65.67 (± 12.30) DSC with I2PC-Net, Fang et al. [105] obtained 79.0% with U-Net based model. Hybrid and transformer-based methods still exposed the limits of NCCT as in the case of Kuang et al. [106] who reported 46.74% DSC and Kirillov et al. [47] with 47.0% DSC for the 3D Swin UNETR. Progresses in segmentation were still modest. Chabi et al. [115] used a 2D U-Net and reported 65.59% DSC on the ATLAS v2.0 dataset, while the DAP-Net approach on CT yielded 56.6% DSC on private dataset which has 706 cases [113].

The vessel segmentation area gained more awareness and the SAM-driven method gave a 97.93% DSC on CTA, which serves as an example of the expanding interest in segmentation tasks that focus anatomically [116]. Kandpal et al. [108] reported 45.0% (± 32.0) DSC. Sony et al. [112] presented an innovative task of synthesizing CBV maps from NCCT, achieving 76.0% DSC with an Attention U-Net. Wang et al. [110] introduced VB-Net for combined prognosis prediction and segmentation on NCCT. Kim et al. [111] achieved 62.2% DSC for Hyperdense Artery Sign (HAS) segmentation using a 2D U-Net ensemble. Liu et al. [59] achieved a high score of 80.3% (± 13.4) DSC for CT segmentation with DCTP-Net. Focusing on CT Perfusion (CTP), Alirr [37] utilized the ISLES 2018 dataset, reporting 59.0% DSC with an Attention ResUnet.

Beyond precise boundary delineation, a substantial volume of the literature focuses on the immediate diagnostic requirements of the acute stroke workflow. These rapid diagnostic tasks (including stroke presence classification, hemorrhage exclusion, and large vessel occlusion (LVO) detection for emergency triage) are detailed in Table 5.

**Table 5:** Deep learning models for CT-based stroke classification, detection, and triage

Reference	Year	Modality	Dataset	Dataset-Size	Model	Performance Metrics				
						ACC	AUC	*P*	Sp	Re
[117]	2025	CT	Private	5772 images	CNN-based	72.0	NR	NR	NR	NR
[118]	2024	CT	Private	4982 images	Hybrid	94.60	NR	95.0	NR	NR
[119]	2024	CT	Kaggle Stroke prediction	249 cases	Sub-clustering based	NR	NR	98.0	NR	95.0
[120]	2024	CT	Private	39 cases	MSE-VGG	90.17	NR	NR	89.86	90.41
[121]	2024	CT	Private	NR	CNN-XGBoost	87.0	NR	NR	NR	97.6
[122]	2024	CT	Private	NR	ENSNET	98.86	98.74	97.71	NR	98.46
[123]	2024	CT	Private	NR	MobileNetV2	92.22	92.0	83.0	NR	92.0
[124]	2025	CT	Private	2000 images	VGG16-based	98.89	NR	98.85	NR	98.93
[103]	2024	CT	Private	100 cases	YOLOv4	99.37	NR	NR	91.14	99.41
		CT	Private	100 cases	SSD	89.92	NR	NR	87.71	86.34
		CT	Private	100 cases	YOLACT	99.30	NR	NR	90.50	99.53
[104]	2024	NCCT	Private	197 cases	I2PC-Net	94.74	94.44	NR	NR	NR
[105]	2024	CT	Private	90 cases	U-Net based	90.00	NR	83.0	100	NR
[14]	2024	NCCT	Private	50 AIS hasta	Hybrid	90.0	NR	NR	86.0	86.0
[125]	2024	NCCT	Private	490 cases	HE-mind	73.3	84.9	NR	90.5	64.1
[126]	2024	CTA	IACTA-EST	301 cases	DRDCNet-3D	NR	91.0	100	100	100
[48]	2024	3D CTA	Private	440 cases	3D ResNet-based	NR	86.9	NR	85.4	78.6
[127]	2024	4D CTA	Private	209 cases	HRNet-based	90.2	94.2	NR	93.5	85.7
[128]	2025	CT	BrSCTHD-2023	770 images	Hibrit ViT-LSTM	94.55	NR	93.0	93.0	93.0
		CT	Kaggle	2501 images	Hibrit ViT-LSTM	96.61	NR	95.0	95.0	95.0
[129]	2025	CT	Private	2023 images	ETC/LGBM	99.51	NR	92.25	95.45	95.45
[130]	2025	CT	Private1	366 cases	VANet	94.54	96.85	96.40	94.53	94.53
		CT	Private2	81 cases	VANet	94.54	91.11	91.80	93.33	93.33
[116]	2025	CT	Private	105 cases	SAM-VM	97.93	NR	98.02	NR	90.65
[131]	2025	CT	Brain Stroke Images Dataset	2501 images	Hibrit CNN-based	98.63	NR	98.76	NR	99.30
[132]	2025	CT	Kaggle Multi	2700 images	DFE-OFS	98.73	NR	98.71	NR	98.5
[109]	2025	NCCT	Private	445 images	Ensemble Model	NR	93.7	NR	88.3	87.8
[133]	2025	CT	Brain Stroke CT Image Dataset	2501 images	CNN-Based	89.73	NR	99.0	NR	96.0
[31]	2025	CT	TEKNOFEST	6951 images	ConvNeXt_Tiny	98.0	NR	99.0	99.0	98.0
[40]	2025	CT	Brain Stroke CT Image Dataset	2501 images	YOLOv9 based	98.7	NR	99.92	NR	99.1
[54]	2025	CT	Private	6951 images	YOLOv9-Seg	NR	NR	99.59	NR	98.93
[61]	2026	CT	Brain Stroke CT Image Dataset	1551 images	EHTO_DKN	90.68	NR	NR	89.69	91.44

*NR *not reported

Beyond precise boundary delineation, a substantial volume of the literature focuses on the immediate diagnostic requirements of the acute stroke workflow. The analysis of ischemic lesions in CT imaging performed by Fontanella et al. [117] saw a revival of sorts, as they used a CNN strategy and achieved 72% accuracy as their results. The upcoming analysis was about more developed predictive modeling. Sharma et al. [118] evaluated a set of hybrid pipelines which combined traditional machine-learning methods and deep-learning modules, and the best-performing combination gave 94% accuracy along with 95% precision. Additionally, the clinical decision-support tasks were analyzed, with Ceskoutsé et al. [119] indicating that their study could predict drug class for ischemic stroke treatment with 98% precision and 95% recall scores.

The identification of LVO through CTA was one of the leading research areas in 2024. It can be said that had a major impact on this field thanks to their implementation of a 3D ResNet architecture which ultimately obtained an AUC of 86.9% [48]. Similar levels of performance were achieved in the evaluation of temporal CTA; Peng et al. [127] got 81.3% AUC and Chatterjee et al. [126] one of the top scores with 91.0% AUC for DRDCNet-3D. NCCT-related research during the same time period targeted various clinical objectives. Zhou et al. [125] have presented their work predicting hematoma enlargement with an 84.9% AUC. Anne and Gueye [121] made use of CNN-generated features along with XGBoost and they managed to get 87% of accuracy. Accuracy of 98.86% was the mark set in ENSNET, which was also very high classification performance [122]. Furthermore, Abumihsan et al. [124] evaluated a VGG16-based model on a private dataset comprising 2000 images, reporting a high ACC of 98.89%. In contrast, Shinohara et al. [14] could claim a 90% accuracy using Dual-Energy CT.

Up to the years of 2025 and 2026, hybrids and attention-driven classifiers had been a reliable source of consistently high performance. Hossain et al. [128] revealed this development with a ViT-LSTM combination that yielded 94.55% accuracy. Inamdar et al. [129] introduced a dual-attention model that not only matched the previous accuracy but also surpassed others by reaching 99.51%. By means of VANet, Kuang et al. [130] brought this movement to LVO detection and realized 94.54% accuracy. Classification tasks continued to show near-perfect accuracy, as seen in PrabhakaraShetty et al. [131] 98.63% ACC, Abumihsan et al. [132] 98.73% ACC.

Beyond pure detection, DL has also been applied to predict complications such as hemorrhagic transformation (HT) following reperfusion therapy. An ensemble model integrating NCCT-based DL features with clinical data achieved an AUC of 93.7 for HT prediction, significantly outperforming traditional clinical scores and providing vital decision support in the emergency department [109]. The YOLO architecture continued to be used successfully in detection tasks by StrokeYOLO [40] − 98.7% ACC and YoloV9-Seg [54] − 98.93% Recall. Finally, studies from 2026 continued the trend of optimized classification models, with 90.68% ACC by EHTO_DKN [61] and 51.6% DSC by a DINOv3 [114], ViT-based models on AIS dataset.

## Discussion

### Datasets

**Fig. 4:**
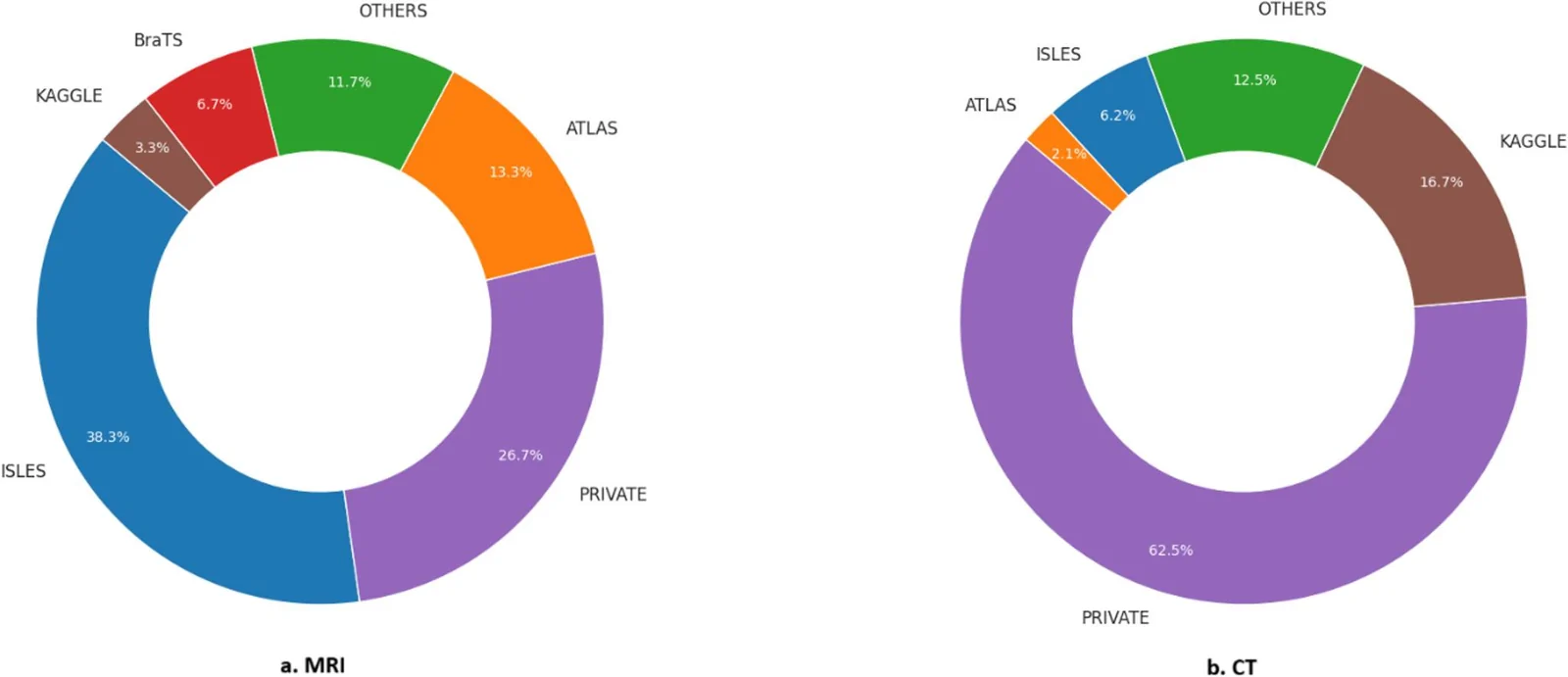
Dataset distribution of the included studies, illustrating the strong reliance on public benchmarks (e.g., ISLES) for MRI research versus the predominance of private datasets in CT studies

MRI-based ischemic stroke segmentation studies have a significant presence in the literature, particularly due to the clarity of lesion boundaries and tissue contrast. The analyzed articles demonstrate clear trends in this area. The vast majority of studies rely on publicly available reference datasets to train and compare models (Fig. 4).

From the analysis of the MRI dataset distribution, it is evident that there is an acute dependency on standardized scholarly benchmarks. The ISLES (Ischemic Stroke Lesion Segmentation) series dominates the list as the primary framework, with the largest percentage of studies at 38.3%. The second-largest group is comprised of private or in-house hospital datasets, which make up 26.7%. It is also evident that the ATLAS (13.3%) and BraTS (6.7%) datasets make up 20% of the total MRI-based studies. The high ranking of the BraTS dataset, which is essentially a brain tumor dataset, is an indicator of the widespread use of transfer learning-based approaches for overcoming the scarcity of data in ischemic stroke research. The use of Kaggle datasets is minimal (3.3%), as depicted in Table 6.

**Table 6:** Datasets used in MRI/CT studies

DATASETS	MRI	CT
PRIVATE	18	34
ISLES	20	3
ATLAS	8	1
KAGGLE	2	7
BraTS	4	0
OTHERS	3	2

In the context of the CT datasets, the overall trend is vastly different. The overall trend is dominated by non-public data. The majority of the studies based on the CT modality (62.5%) have utilized private, in-house, or institutional datasets. This is essentially the exact opposite of the overall trend based on the MRI modality. The use of the Kaggle datasets is far more prevalent (16.7%) for the CT modality than the MRI modality. The overall trend is also dominated by the reduced use of the overall academic benchmarks, specifically the ISLES 2018 CTP challenge and the ATLAS datasets, which are collectively utilized in only 8.3% of the overall studies.

ISLES datasets (e.g., ISLES 2015, 2017, 2022 versions) serve as the de facto standard in the field. A significant number of studies have validated their model’s performance on this benchmark [7, 28, 55, 56, 71, 75, 76, 78, 81, 85, 89, 93]. The ATLAS (v1.2, v2) dataset, which notably includes chronic lesions, has also been widely utilized [39, 55, 60, 73, 74, 78, 82, 94]. Interestingly, many studies have utilized the BraTS tumor segmentation dataset for the purpose of “transfer learning” to compensate for the scarcity of ischemic stroke data [39, 75, 94, 134]. A considerable number of studies have used private, in-house datasets [56, 64, 73, 79, 97, 135]. Or datasets obtained from platforms such as Kaggle [58]. However, a critical synthesis of these datasets reveals an inverse relationship between dataset complexity and reported performance. Early benchmarks like ISLES 2015 often yield artificially high DSC scores (> 85%) due to their small size and homogeneity. In contrast, larger, multi-center datasets like ISLES 2022 present a more realistic challenge with diverse scanner protocols, resulting in lower but more generalizable performance metrics. Therefore, high scores on limited private datasets should be interpreted with caution, as they often reflect lower task complexity rather than superior model generalization.

### Models

Significant research has been conducted on the segmentation of ischemic stroke lesions, resulting in the development of various DL architectures. Unlike conventional machine learning methods, DL models have been found to perform better in learning hierarchical features from data. Notable is the fact that most of the models are variants of the U-Net and CNN architecture (Table 7).

**Table 7:** Model used in MRI/CT studies. (The “Others” category includes less common or specific architectures such as GAN, Capsule Networks, Spiking Networks, EHTO_DKN, SAM-VM, HE-mind, and FTL)

MODEL FAMILY	MRI	CT
U-Net-based	20	7
CNN-based (non-U-Net)	24	13
Transformer/Hybrid-based	7	9
ML-based/Ensemble	1	9
YOLO-based	0	5
Others (New/Specific Architectures)	8	5

**Fig. 5:**
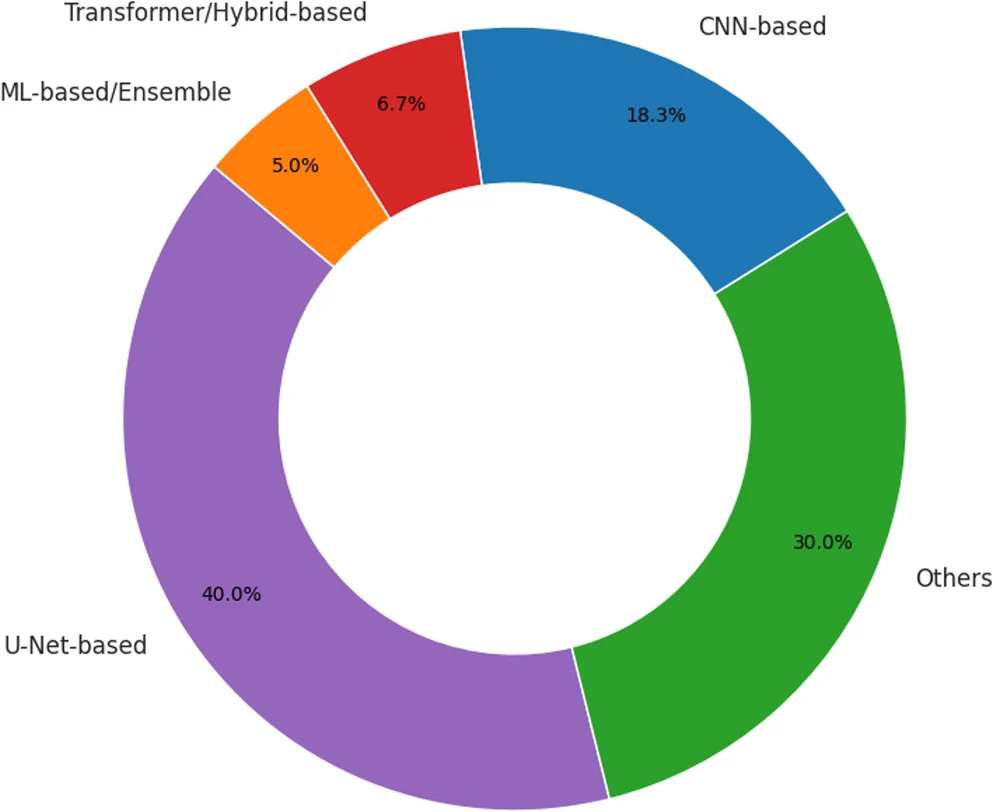
Distribution of deep learning architectures utilized in MRI-based studies, highlighting the clear dominance of U-Net variants for precise segmentation tasks

The distribution of model architectures utilized according to the results from the analyzed MRI articles is presented in Fig. 5.

#### U-Net-based (40.0%)

This is the most dominant architecture in the MRI literature. The vast majority of studies were based on U-Net architectures, such as 3D U-Net [97], U-Net + ResNet [64], ResU-Net [43, 93], Cascade ResU-Net [79], Attention U-Net [85], and MRA-UNet [56]. U-Net and its variants (e.g., Attention, ResUnet, nnU-Net, TransUNet) are by far the most popular approach for MRI segmentation. This dominance is not merely a trend but a result of architectural suitability. Our analysis suggests that incorporating attention mechanisms (e.g., Attention U-Net) generally yields a consistent performance gain over standard architectures. This is likely because attention gates actively suppress the background noise inherent in medical scans, allowing the model to focus on irregular lesion boundaries. Similarly, hybrid Transformer models are beginning to outperform pure CNNs in large-lesion scenarios by capturing long-range global dependencies that local convolutions often miss.

#### Others (30.0%)

The second largest group comprises specific or novel architectures, including MSCS-Net [94], and Deep Spiking MobileNet (DSpM-Net) [58]. This indicates that the search for alternatives to U-Net is also a strong research trend.

#### CNN-based (18.3%)

General CNN-based architectures other than U-Net (e.g., ResNet, DenseNet, VGG-based models) rank third.

#### Transformer/Hybrid-based (6.7%)

Pure Transformer or Transformer-heavy hybrid models appear to have found less application in MRI compared to CT.

#### ML-based/Ensemble (5.0%)

Architectures based on classical machine learning or ensemble models are the least utilized group in the MRI literature.

**Fig. 6:**
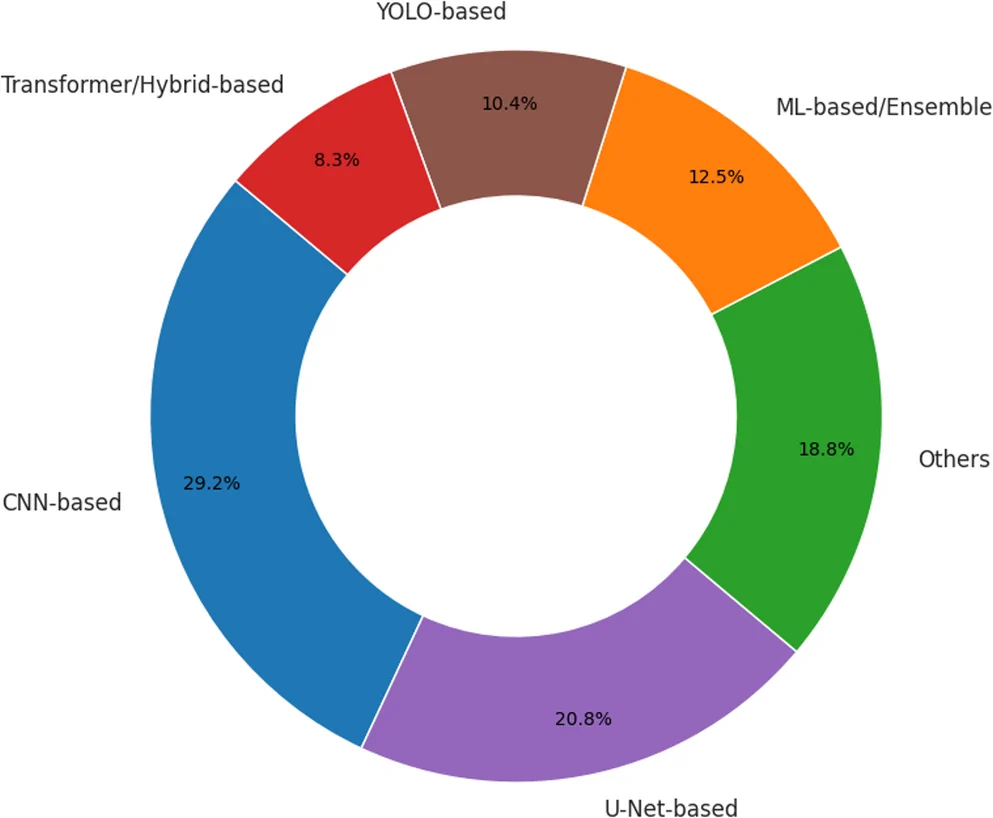
Distribution of deep learning architectures utilized in CT-based studies, reflecting a broader algorithmic diversity (including YOLO and general CNNs) tailored for rapid classification and triage

The distribution of model architectures used, based on the results from the analyzed CT articles, is presented in Fig. 6.

#### CNN-based (29.2%)

This is the most common category in CT studies, comprising general CNN architectures rather than U-Net variants. This is primarily because classification and detection tasks (e.g., VGG or ResNet-based) are highly prevalent in CT applications.

#### U-Net-based (20.8%)

U-Net variants constitute the second most popular group for CT segmentation.

#### Others (18.8%)

Novel and specific architectures (e.g., SAM-VM, EHTO_DKN, HE-mind, FTL) represent a research area in CT that is as active as it is in MRI.

#### ML-based/Ensemble (12.5%)

Classical ML and hybrid models (e.g., CNN-XGBoost, Ensemble) have been used much more frequently in CT studies compared to MRI.

#### YOLO-based (10.4%)

The YOLO architecture, which is focused on rapid detection, has found a distinct application area in CT, aligning with its suitability for emergency department use cases (rapid detection), which is not as prominent in MRI literature.

#### Transformer/Hybrid-based (8.3%)

Transformers are also utilized in CT studies at a rate similar to that observed in the MRI literature.

**Fig. 7:**
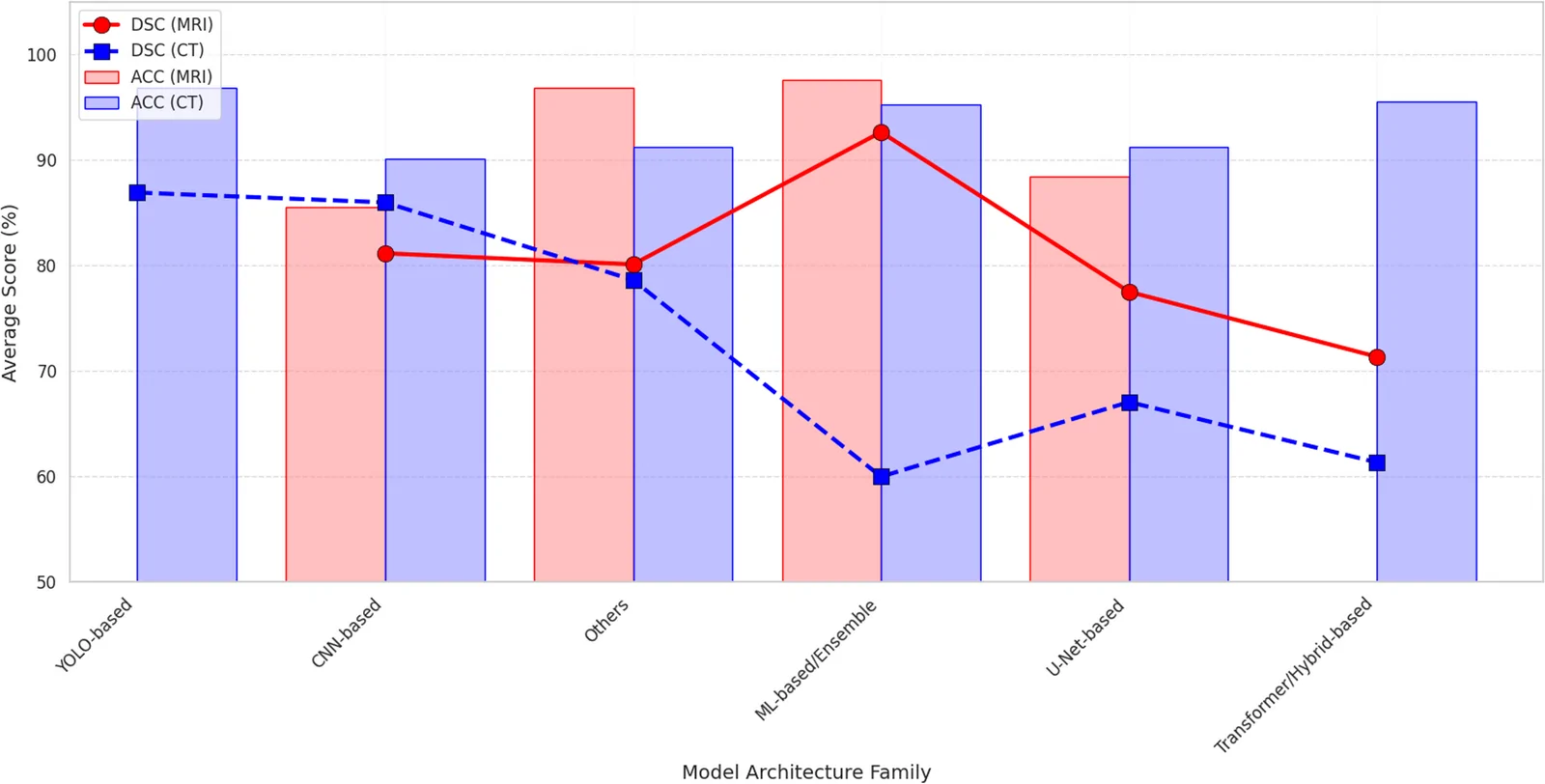
Average DSC (Line) and ACC (bar) performance by model family and modality. Average MRI DSC metric show as red-line, ACC metric as red-bar, CT DSC metric as blue-line and ACC metric as blue-bar in this fig. The “Transformer/Hybrid-based” category for MRI (red) lacks and ACC bar as no studies in this specific group reported an average ACC score

The most prominent pattern in the graph (Fig. 7) is that the CT (blue) bars (ACC) are almost entirely above 90%, whereas the blue lines (DSC) remain much lower (in the 60–80% range). This strongly indicates that while CT-based models are highly successful in classification tasks (e.g., “stroke present/absent” or “is this pixel healthy?“), they struggle significantly with delineating the precise lesion boundaries (DSC).

For the MRI (red) data set, the DSC curve, especially for the “ML-based/Ensemble,” “U-Net-based,” and “CNN-based” groups, is close to or even crosses the ACC bars. This shows that the MRI models, especially those based on Ensemble, U-Net, and CNN architectures, are not only accurate (in terms of ACC), but their DSC is large as well, implying their reliability for the segmentation task.

#### ML-based/Ensemble

This category demonstrates some of the highest average DSC scores for both MRI (92.6% DSC) and CT (highest in MRI). This indicates that combining multiple models (ensembles) significantly improves segmentation accuracy.

#### U-Net-based

This remains the most stable and reliable segmentation architecture for both MRI (77.5% DSC) and CT (67.0% DSC).

#### YOLO-based

As expected, this category (present only in CT) shows high ACC (96.8%) but, compared to its DSC (86.9%), confirms its strength lies more in detection than segmentation. (It is noteworthy, however, that its DSC is still above the CT average).

#### Transformer/hybrid-based

This category lags slightly behind U-Net-based models for both MRI (71.3% DSC) and CT (61.3% DSC). This may suggest that Transformer-based models are still in the optimization phase or that they struggle with low-contrast data, such as NCCT.

MRI-based segmentation, owing to the high intrinsic contrast of DWI and FLAIR, generally yields high DSC values. de la Rosa et al. [17] achieved an exceptional DSC score of 94.0% on the ISLES 2022 dataset with an ensemble model. Similarly, Cinar et al. [85] reported 94.86% DSC on the same dataset with an Attention U-Net-based model. Many other studies also reported strong DSC scores above 80%: Sathish & Sheet [93] with 87.7% on ISLES 2015 using ResUNet-DSM; Rahman et al. [89] with 87.49% on ISLES 2022 with a DenseNet-based model; Liu et al. [86] with 86.63% on ISLES 2022 with an InceptionNeXt based model on private datasets. Alongside segmentation, ACC metrics in classification tasks (stroke presence/absence or type) are also quite high. For example, Patil & Govindaraj [96] achieved 95.67% ACC with ResNet50; Bhuvana & Hemalatha [98] reported 97.80% ACC with a custom model; and Smitha et al. [99] achieved 99.3% ACC on fMRI data.

Some studies have directly compared the impact of different MR sequences on performance. For instance, Sinha et al. [83], using their EnigmaNet model on the ISLES 2015 dataset, achieved a higher DSC on the FLAIR sequence (89.65%) compared to the DWI sequence (84.23%). This indicates that different sequences may offer advantages depending on the model architecture and the task (acute vs. subacute). Other studies, such as Nazari-Farsani et al. [136], have focused specifically on acute lesion detection using DWI. CT is the preferred imaging method in emergency diagnostics due to its speed and accessibility. However, the low contrast of ischemic lesions, especially on NCCT images, makes segmentation a more challenging task compared to MRI.

Unlike the MRI analysis, a significant density of private, in-house datasets is observed in CT-based studies [54, 104–106, 110, 111, 116, 118, 130]. Nonetheless, public datasets such as Kaggle, TEKNOFEST, ISLES 2018 (for CTP data), and ATLAS v2.0 have also been utilized. Segmentation-focused architectures such as SwinUNETR, 3D U-Net, Attention ResUnet, U-Net++, Hybrid CNN-Transformer, and DCTP-Net are present. Models like YOLO and its variants, Hybrid ViT-LSTM, MobileNetV2, CNN-XGBoost, and 3D CNN are generally used for “stroke presence/absence” or “ischemic/hemorrhagic” classification.

The reviewed studies show that performance must be interpreted in relation to both imaging modality and task type. CT-based models often report high accuracy or AUC for image-level or patient-level tasks such as stroke detection, hemorrhage exclusion, or large-vessel occlusion identification. These results, however, should not be directly compared with Dice or IoU values reported in segmentation studies. Classification metrics measure whether a model can distinguish between diagnostic categories, whereas segmentation metrics measure how accurately the model localizes infarcted tissue. This distinction is especially important for NCCT, where early ischemic changes are subtle and lesion margins are often poorly defined. Therefore, CT-based deep learning appears more developed for triage-oriented detection than for precise voxel-level lesion segmentation.

Conversely, in the MRI data, the DSC (segmentation) line is much closer to the ACC bars. This demonstrates that MRI models, particularly those in the ML-based/Ensemble, U-Net-based, and CNN-based categories, exhibit not only high classification accuracy but also high spatial overlap (DSC) success, implying they are more reliable for the segmentation task than their CT counterparts. MRI-based segmentation, owing to the high intrinsic contrast of DWI and FLAIR sequences, generally yields high DSC values. State-of-the-art (SOTA) studies have reported scores exceeding 94% on the ISLES 2022 dataset: de la Rosa et al. [17] achieved an exceptional DSC score of 94.0% with an ensemble model, and Cinar et al. [85] reported 94.86% DSC on the same dataset with an Attention U-Net-based model.

Beyond these peak scores, numerous other studies have reported strong DSC scores above 80%: Sathish & Sheet [93] with 87.7% on ISLES 2015 using ResUNet-DSM; Rahman et al. [89] with 87.49% on ISLES 2022 with a DenseNet-based model. Alongside segmentation, ACC metrics in classification tasks (stroke presence/absence or type) are also quite high. For example, Patil & Govindaraj [96] achieved 95.67% ACC with ResNet50; Bhuvana & Hemalatha [98] reported 97.80% ACC with a custom model; and Smitha et al. [99] achieved 99.3% ACC on fMRI data.

Some studies have directly compared the impact of different MR sequences on performance. For instance, Sinha et al. [83], using their EnigmaNet model on the ISLES 2015 dataset, achieved a higher DSC on the FLAIR sequence (89.65%) compared to the DWI sequence (84.23%). This indicates that different sequences may offer advantages depending on the model architecture and the task (acute vs. subacute).

CT’s role in the emergency department is reflected as “task diversity” in the literature. Classification/detection tasks appear much more mature compared to segmentation. Studies have reported exceptional ACC rates between 98 and 99% for “stroke presence/absence” classification [122, 129, 132]. However, the task of delineating the exact lesion boundaries (segmentation) is much more difficult in CT and shows significant variation depending on the modality:

#### CTA (angiography)

The task is generally LVO detection or vessel tree segmentation. High scores such as 90.63% DSC [116] and high AUC values for LVO detection [126] (91.0% AUC) have been achieved in this area.

#### CTP (perfusion)

This is generally used for “core” and “penumbra” segmentation. Studies achieved ~ 76.0% DSC on CTP data, indicating higher success than NCCT [37, 112].

#### NCCT (non-contrast CT)

The most challenging segmentation task (with low DSC scores) is concentrated in this modality. Studies about 46.7%-47.0% DSC confirm this difficulty [47, 102]. SOTA results on NCCT were achieved by Kuang et al. [106] with I2PC-Net (77.97% DSC).

A deeper discussion of this performance gap reveals that it is driven more by pathophysiology than by algorithmic limitations. NCCT exhibits low sensitivity in the early hours of stroke because the tissue density changes (hypodensity) indicative of ischemia have not yet fully developed. In sharp contrast, DWI detects cytotoxic edema—the restriction of water movement—within minutes of onset. Consequently, the lower segmentation scores in NCCT are largely attributable to the modality’s inherent lack of contrast for early ischemic changes, rather than a failure of the DL architectures themselves.

When the average performance of all the architectures is studied, certain trends are observable. The ML/Ensemble category shows the best average segmentation performance overall, particularly for MRI scans, where the DSC averages 92.6%. This suggests that the combined power of multiple models significantly improves performance. The performance of the U-Net family remains the most stable and dependable workhorse for segmentation tasks on both MRI scans (77.5% average DSC) and CT scans (67.0% average DSC). The performance of the YOLO family, which is present exclusively on CT scans, reinforces the strong detection capacity of this family of models with an ACC of 96.8%, which is coupled with an exceptionally high DSC of 86.9%, significantly outperforming the average performance on CT scans by a large margin. Finally, the Transformer/Hybrid family performs slightly lower than the U-Net family on average for MRI scans (71.3% average DSC) and CT scans (61.3% average DSC). This may indicate that this family of models is still being fine-tuned and perhaps struggles to achieve the expected performance on low-contrast data, such as NCCT scans.

When evaluating the performance of DL models, the choice of metrics reflects the primary goal of the study. Our systematic review reveals an asymmetric distribution of AUC and IoU metrics between the CT and MRI literature. This situation highlights a fundamental difference in research focus, originating from the different roles these two modalities play in the clinical workflow. AUC, the primary classification metric measuring a model’s ability to distinguish between positive and negative classes (e.g., “stroke” vs. “no stroke”), is reported far more frequently and with higher scores in the CT literature. CT models focused on classification, such as Hybrid CNN-based (100%), DFE-OFS (99.0%), and ENSNET (98.74%), have demonstrated near-perfect discriminatory power. This high success is not limited to simple classification; even in complex tasks such as LVO detection (VANet, 96.85%) or penumbra/core segmentation on NCCT (I2PC-Net, 94.44%), AUC scores exceed 90%. These findings indicate that CT-based detection and classification models have reached promising retrospective performance in selected datasets, but they should not yet be regarded as solved clinical tools. Many studies still rely on single-center data, private datasets, retrospective labels, and limited external validation. In MRI-based studies, the lower emphasis on AUC reflects a different research question. Rather than asking only whether stroke is present, many MRI studies focus on where the lesion is, how large it is, and how accurately its borders can be defined. Although DWI and ADC often make ischemic lesions more visible than NCCT, small infarcts, boundary uncertainty, scanner variation, and reader-dependent annotations remain important challenges.

Whereas the IoU (Jaccard Index) metric, a more rigorous segmentation evaluation method than the DSC metric, reverses this trend by being more frequently cited in MRI studies than CT studies. In fact, state-of-the-art MRI segmentation models, including DenseNet-based models (84.63%) and MSCS-Net (82.81%), consistently report IoU values of more than 80% on standard datasets including ISLES 2022. An IoU above 0.80 indicates strong spatial overlap under the conditions of a given dataset and annotation protocol. It should not be interpreted as near-perfect clinical performance. In practice, segmentation reliability is affected by lesion size, lesion location, imaging quality, scanner differences, preprocessing choices, and inter-reader variability. For this reason, high IoU values on benchmark datasets need to be supported by external validation before they can be considered clinically reliable. By contrast, IoU values in CT image segmentation are limited in number and of dual nature: although IoU values of more than 80% are reported in high-contrast image segmentation, including CTA vessel segmentation by the state-of-the-art SAM-VM model (83.54%) and CTP segmentation by DCTP-Net (79.0%), the results are far less impressive in the most critical segmentation task in CT image segmentation: NCCT segmentation, in which IoU values of less than 50% are reported by hybrid models including Hybrid CNN-ViT (CFIN + BDL) (43.3%), HUT-NSS (49.5%), and DINOv3-ViT-based (51.0%), signifying the most significant challenge in image segmentation in the existing literature.

**Fig. 8:**
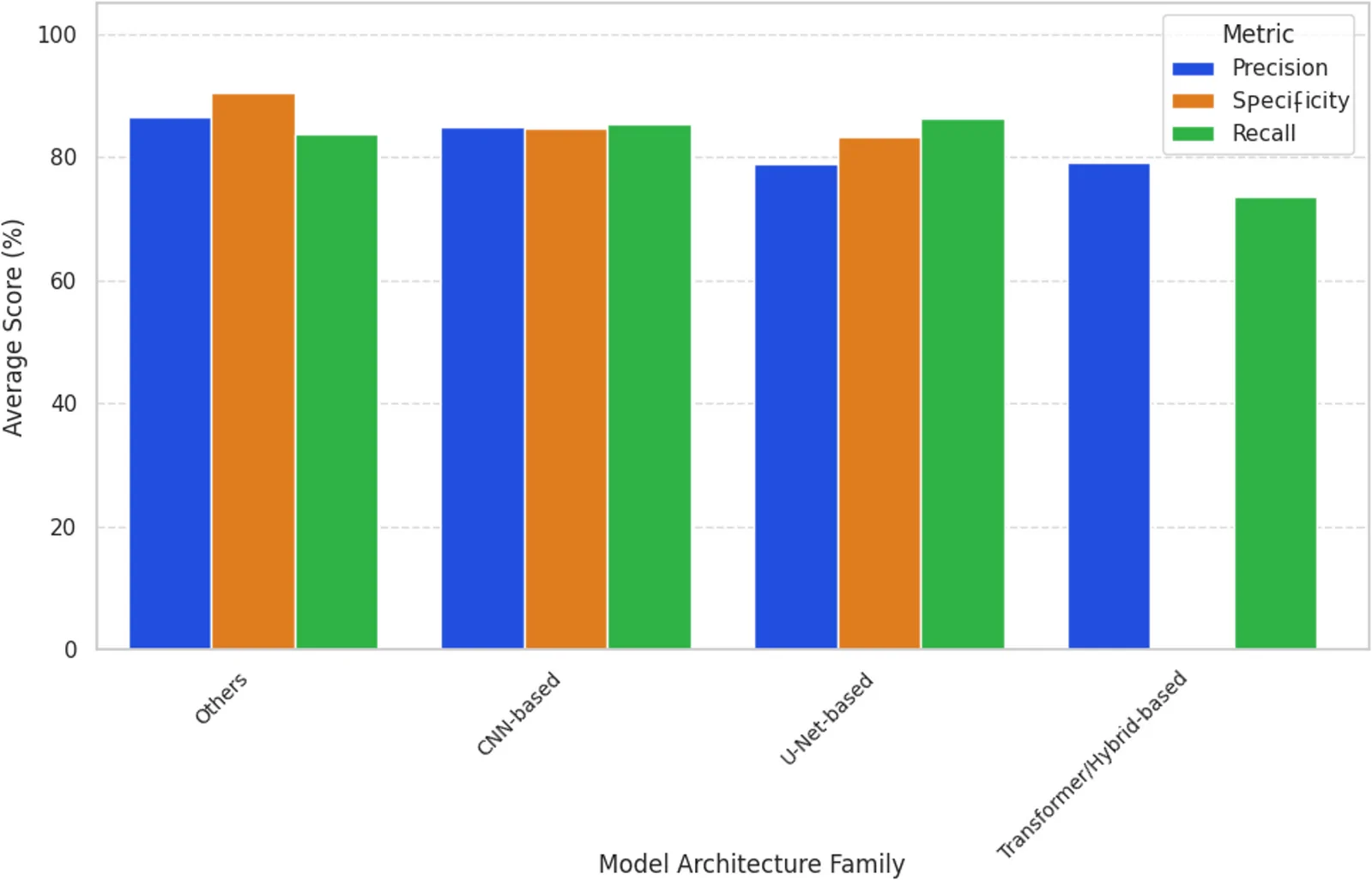
Average precision, specificity, and recall by model family (MRI Modality)

The clinical performance profile of MRI-based models is illustrated in Fig. 8. In general, established architectures such as U-Net-based, CNN-based, and Transformer/Hybrid-based models demonstrate a relatively balanced performance across all three metrics (Precision, Specificity, and Recall), typically around the 80% level. This suggests that these models achieve a reasonable trade-off between “false alarms” (low Precision) and “missing lesions” (low Sensitivity/Recall). The highest and most balanced bars in the graph belong to the ML-based/Ensemble (e.g., hybrids like GCDCM, DSPM-Net) and Others (e.g., MSCS-Net) categories. The fact that these models achieve average scores above 90% indicates that specially optimized hybrid approaches or novel architectures may be more successful than standard U-Nets at both avoiding missed lesions (high Sensitivity/Recall) and preventing false alarms (high Precision). The Transformer/Hybrid-based category displays a slightly lower and more imbalanced profile compared to U-Net and CNNs (particularly with a lower Recall). This suggests that these models may still be in the optimization phase for MRI applications, or that their comparatively lower performance on specific datasets (e.g., ATLAS) negatively impacts the overall average.

**Fig. 9:**
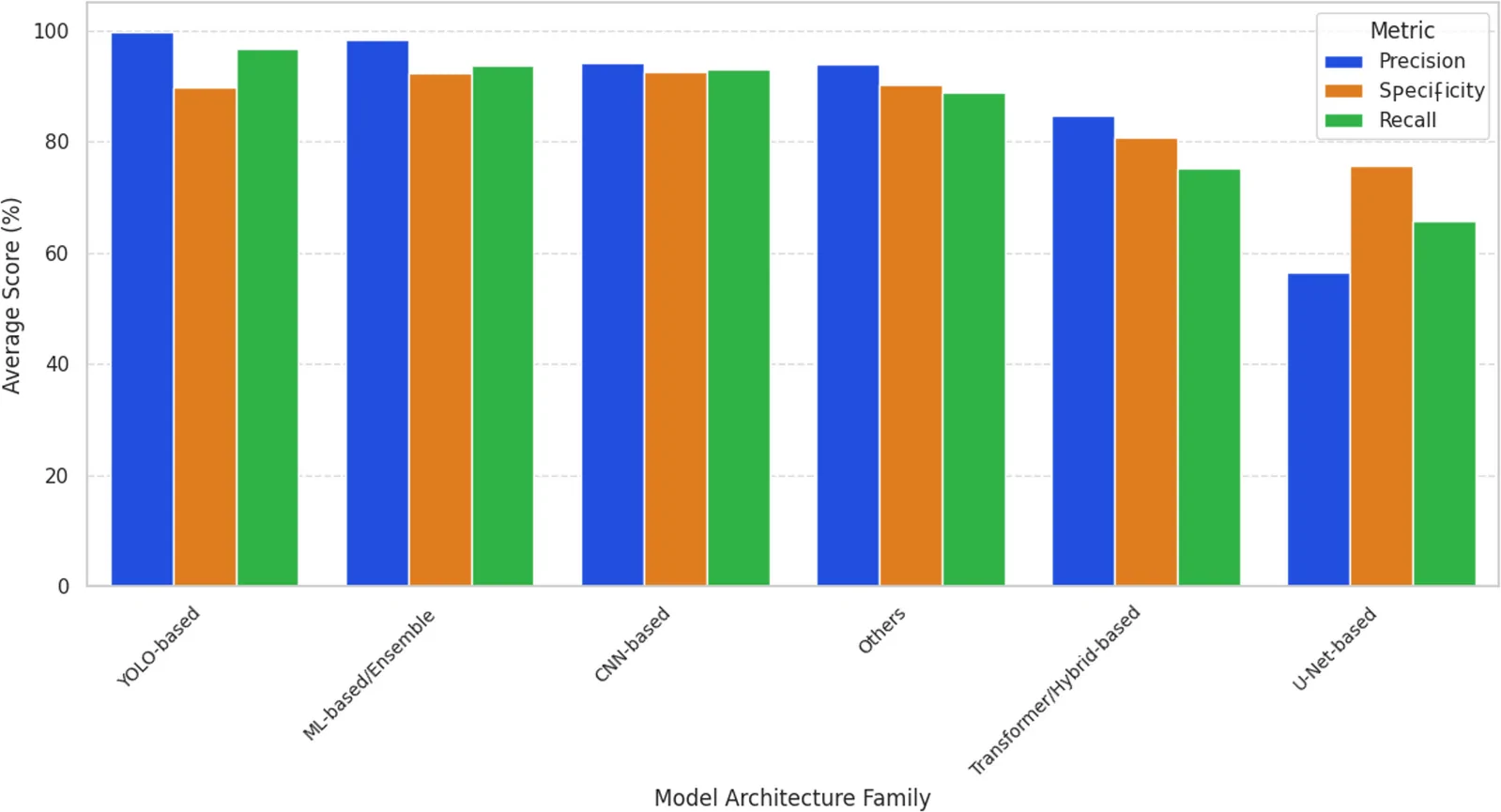
Average precision, specificity, and recall by model family (CT Modality)

The distinct profile of CT-based models compared to MRI is strikingly demonstrated in Fig. 9, clarifying the existence of two different task groups. The bars for the CNN-based, ML-based/Ensemble, and YOLO-based categories are almost entirely above 90%, with some approaching 95%. These extremely high scores clearly show that the majority of the models were used for less complex tasks, specifically classification (presence/absence of stroke) or detection (presence/absence of large vessel occlusion), rather than the more challenging task of segmentation (boundary delineation). In the former, the model’s performance is close to perfection, as exemplified by the DRDCNet-3D model with 100% score, the ConvNeXt_Tiny model with 99% score, and so on.

The most prominent difference between the scores can be seen in the category based on the Transformer/Hybrid model. Here, the average scores, which vary between 40% and 75%, are much lower compared to the scores obtained by the other categories. This difference can be explained by the fact that the models included in this category, such as SwinUNETR, Hybrid CNN-Transformer (CFIN + BDL), were not used for the “classification” task, as was the case with the other categories, but for the much more challenging NCCT segmentation task. This can be confirmed by the low precision/ sensitivity/ recall (P/S/R) scores, which are consistent with the lower DSC scores (46–60%) that were obtained previously. In the category based on the U-Net model, the scores, as might be expected based on the results obtained for the MRI modality, show a more balanced picture, with the scores for the CT modality also converging at the 70–80% level. This can be interpreted as the U-Net model providing a middle-ground performance for both the classification and the slightly easier segmentation tasks (CTP/CTA).

### The “U-Net Bottleneck” and the need for architectural shifts

A critical synthesis of the current literature reveals that a disproportionate amount of research remains anchored to the U-Net architecture or its minor iterative modifications (e.g., the integration of localized attention gates). While foundational, it is increasingly evident that the field has reached a level of saturation with these approaches. Extracting marginal, fractional improvements in DSC through minor structural tweaks offers diminishing returns for real-world clinical applicability. To solve complex neuroradiological challenges, the field must transcend this “U-Net bottleneck.” Future research should prioritize truly novel paradigms that address specific clinical workflows, such as pure ViTs capable of global contextual reasoning for highly subtle lesions, hybrid real-time object detectors for immediate emergency triage, and multi-modal networks that shift the algorithmic focus entirely from spatial delineation to functional outcome prediction.

### Clinical integration and neuroradiology perspectives

While high DSC and AUC scores indicate technical success, the true value of DL in acute ischemic stroke lies in its integration into the neuroradiology workflow. From a clinical perspective, NCCT remains the first-line imaging modality. However, early ischemic changes, such as the loss of the insular ribbon or subtle basal ganglia obscuration, are notoriously difficult to detect due to low tissue contrast. DL models that automate the Alberta Stroke Program Early CT Score (ASPECTS) are proving invaluable in standardizing these subtle NCCT evaluations, effectively reducing inter-reader variability during emergency triage.

Furthermore, the decision for EVT increasingly relies on identifying the ischemic penumbra. DL applications in CT Perfusion (CTP) and MRI Perfusion-Weighted Imaging (PWI) are shifting the paradigm towards automated “perfusion mismatch” assessment. By rapidly delineating the irreversibly damaged infarct core versus the salvageable penumbra, these models directly inform thrombectomy triage, especially for patients presenting in extended time windows or with unknown times of onset.

Ultimately, for these advanced models to transition from the laboratory to the clinic, they must overcome workflow friction. Practical integration requires that AI predictions be seamlessly embedded directly into existing Picture Archiving and Communication Systems (PACS) or Radiology Information Systems (RIS), providing zero-click, automated secondary reads that operate concurrently with the radiologist’s evaluation.

### Limitations

While this systematic review demonstrates the impressive progress of DL in ischemic stroke lesion segmentation, the analysis of the literature reveals that the field still faces significant and unresolved limitations.

A major limitation in current research is what can be termed the Generalizability Crisis and Dataset Scarcity. From the findings, it is evident that most models, especially those analyzing CT scans, have been trained on private, single-center, or in-house data. This over-reliance leads to a significant problem termed domain shift, whereby models trained on data collected in one hospital frequently fail on out-of-distribution data from another due to profound dataset heterogeneity (e.g., variations in scanner manufacturers, slice thicknesses, and reconstruction algorithms). Consequently, direct head-to-head comparisons of performance metrics (such as DSC or AUC) between models evaluated on different datasets must be interpreted with extreme caution. For instance, a model achieving a high Dice score on a small, homogeneous, single-center private dataset may lack the real-world robustness of a model with a slightly lower score that was stringently evaluated on a diverse, multi-center benchmark. Although some MRI benchmarks, such as ISLES and ATLAS, have helped mitigate this somewhat, they still do not fully account for the diversity of the global population and the vast variations of clinical scanners.

Another limitation is the difficulty of NCCT lesion segmentation and the way performance metrics are sometimes interpreted. Early ischemic changes on NCCT are often faint, low-contrast, and difficult to separate from normal tissue variation. This makes voxel-level delineation considerably harder than image-level classification or detection. Some CT-based studies report very high accuracy for classification tasks, but this does not mean that the same models can reliably segment infarct boundaries. Accuracy can also be misleading in imbalanced datasets, particularly when the lesion represents only a small part of the image. For this reason, CT studies should clearly separate image-level detection, patient-level classification, and voxel-level segmentation, and should report metrics that are appropriate for each task.

A third limitation can be defined as “Model Homogeneity and Superficial Novelty.” Indeed, a large body of work on MRI applications is centered on the U-Net architecture or minor modifications of this structure, such as the addition of attention or residual modules. Although these models exhibit state-of-the-art performance, this phenomenon indicates a certain saturation of the field and a need for novel approaches to tackle real-world challenges.

Furthermore, a significant challenge encountered during this synthesis was the lack of standardized reporting for dataset characteristics across the literature. Many studies fail to specify exact sample sizes or the specific subsets utilized from public benchmarks. Additionally, there is a persistent lack of uniformity in reporting units, with studies intermittently using ‘cases’ (referring to 3D patient volumes) or ‘images’ (referring to 2D slices). This inconsistency complicates the direct comparison of model performance relative to data scale and limits the overall interpretability of results across different architectural frameworks.

A further limitation is the gap between technical performance and clinical use. Most studies report metrics such as Dice, IoU, AUC, or accuracy, but fewer studies test whether these outputs improve treatment selection, reporting time, radiologist confidence, or patient-level risk stratification. A high Dice score may show good agreement with a reference mask, but it does not by itself demonstrate clinical value. For neuroradiology practice, future models should be evaluated against endpoints that matter in acute stroke care, including treatment eligibility, infarct growth, hemorrhagic transformation, functional outcome, and recurrence risk. This is why the field is gradually moving from isolated pixel-level analysis toward imaging-based prognostic support.

## Conclusion and future directions

Deep learning research in acute ischemic stroke imaging has reached a stage where technical performance can no longer be interpreted independently of modality, task definition, and clinical workflow. The evidence reviewed in this study shows a clear separation between MRI- and CT-based applications. MRI-based studies, particularly those using DWI, ADC, FLAIR, and multimodal inputs, have produced the strongest results for lesion segmentation and tissue characterization. U-Net-derived architectures, self-configuring frameworks, attention mechanisms, and selected CNN-Transformer hybrids have improved spatial overlap metrics on public benchmarks. However, high Dice or IoU values on curated datasets should be interpreted as evidence of technical feasibility rather than clinical readiness. Robust deployment requires external validation, patient-level data separation, scanner-level generalizability, transparent annotation standards, and clinically meaningful error analysis.

CT-based studies reflect a different clinical priority. In emergency stroke care, CT is used primarily for rapid triage, hemorrhage exclusion, large-vessel occlusion assessment, ASPECTS-related interpretation, and perfusion-based treatment selection. Many CT models report high retrospective accuracy or AUC for classification and detection tasks, but these values should not be equated with reliable lesion boundary delineation. This distinction is particularly important for NCCT, where early ischemic changes are subtle, low-contrast, and variably visible even to experienced readers. Future CT studies should therefore define the intended clinical task more precisely and avoid mixing image-level classification, patient-level detection, perfusion estimation, and voxel-level segmentation under the same performance narrative.

The main barrier for the field is no longer the absence of model architectures, but the lack of rigorous clinical validation. Many recent studies still depend on private, single-center, retrospectively curated datasets, and reporting remains inconsistent regarding sample size, lesion burden, acquisition protocol, annotation process, and external testing. Future work should place less emphasis on marginal architectural modifications and more emphasis on reproducibility, multicenter evaluation, prospective testing, calibration, interpretability, and integration with neuroradiology workflow. Models should also be tested in clinically difficult scenarios, including small infarcts, posterior circulation strokes, motion-degraded scans, low-volume lesions, discordant CT-MRI findings, and cases with uncertain treatment eligibility.

The next stage of deep learning in acute ischemic stroke imaging should move from isolated pixel-level prediction toward prognosis-aware and decision-oriented systems. Clinically useful models should support treatment selection, infarct growth estimation, hemorrhagic transformation risk assessment, functional outcome prediction, recurrence risk stratification, and reporting efficiency. This requires models that combine imaging features with clinical variables, temporal information, and outcome data, while remaining interpretable and compatible with PACS/RIS-based workflows. Deep learning will have meaningful value in neuroradiology only when strong benchmark performance is linked to reliable, explainable, and externally validated support for real-time stroke decision-making.

## Data Availability

All data analyzed in this review were extracted from previously published articles and publicly available datasets cited in the manuscript. The data extraction sheets and summary tables generated during the study are available from the corresponding author, on reasonable request.

## Declarations

### Use of artificial intelligence tools

During manuscript preparation, AI-based language assistance was used only to improve grammar, readability, and editorial clarity. No AI tool was used to generate study data, perform study selection, extract results, conduct quality assessment, interpret findings, or draw scientific conclusions. All intellectual content, methodological decisions, interpretations, and final revisions were reviewed and approved by the authors.
